# A promoter–RBS library for fine-tuning gene expression in *Methanosarcina acetivorans*

**DOI:** 10.1128/aem.01092-24

**Published:** 2024-08-12

**Authors:** Ping Zhu, Mariana Molina Resendiz, Ingemar von Ossowski, Silvan Scheller

**Affiliations:** 1 Department of Bioproducts and Biosystems, School of Chemical Engineering, Aalto University, Espoo, Finland; University of Nebraska-Lincoln, Lincoln, Nebraska, USA

**Keywords:** promoter, RBS, library construction, 5'UTR-engineering, gene expression, methanogens, *Methanosarcina acetivorans*

## Abstract

Methanogens are the main biological producers of methane on Earth. *Methanosarcina acetivorans* is one of the best characterized methanogens that has powerful genetic tools for genome editing. To study the physiology of this methanogen in further detail as well as to effectively balance the flux of their engineered metabolic pathways in expansive project undertakings, there is the need for controlled gene expression, which then requires the availability of well-characterized promoters and ribosome-binding sites (RBS). In this study, we constructed a library of 33 promoter–RBS combinations that includes 13 wild-type and 14 hybrid combinations, as well as six combination variants in which the 5'-untranslated region (5'UTR) was rationally engineered. The expression strength for each combination was calculated by inducing the expression of the β-glucuronidase reporter gene in *M. acetivorans* cells in the presence of the two most used growth substrates, either methanol (MeOH) or trimethyl amine (TMA). In this study, the constructed library covers a relatively wide range (140-fold) between the weakest and strongest promoter–RBS combination as well as shows a steady increase and allows different levels of gene expression. Effects on the gene expression strength were also assessed by making measurements at three distinct growth phases for all 33 promoter–RBS combinations. Our promoter–RBS library is effective in enabling the fine-tuning of gene expression in *M. acetivorans* for physiological studies and the design of metabolic engineering projects that, e.g., aim for the biotechnological valorization of one-carbon compounds.

**IMPORTANCE:**

Methanogenic archaea are potent producers of the greenhouse gas methane and thus contribute substantially to global warming. Under controlled conditions, these microbes can catalyze the production of biogas, which is a renewable fuel, and might help counter global warming and its effects. Engineering the primary metabolism of *Methanosarcina acetivorans* to render it better and more useful requires controllable gene expression, yet only a few well-characterized promoters and RBSs are presently available. Our study rectifies this situation by providing a library of 33 different promoter–RBS combinations with a 140-fold dynamic range in expression strength. Future metabolic engineering projects can take advantage of this library by using these promoter–RBS combinations as an efficient and tunable gene expression system for *M. acetivorans*. Furthermore, the methodologies we developed in this study could also be utilized to construct promoter libraries for other types of methanogens.

## INTRODUCTION

As methane producers, *Methanosarcina* species have gained attention due to their well-characterized genomes (1–3) and diverse metabolisms (4). *Methanosarcina acetivorans* stands out as an ideal genetic workhorse and model organism for studies involving basic research and biotechnological applications. This has intensified with the development of self-replicating *Escherichia coli–Methanosarcina* shuttle vectors for gene expression, these being derived from the *M. acetivorans* endogenous pC2A plasmid (5). The adoption of φC31 integrase-mediated recombination in *Methanosarcina* has further provided an efficient tool for chromosomal integration of heterologous genes (6). With the recent development of CRISPR genetic tools, genome editing for metabolic engineering applications in *Methanosarcina* species has gathered momentum and sped forward (7, 8).

Utilization of metabolic engineering approaches typically requires that gene expression is controlled in host cells to balance the metabolic flux and optimize biomass production. A suitable genetic tool for fine-tuning gene expression becomes important. For this, an efficient approach is to engineer the promoter and/or RBS, which would regulate the gene expression at both the transcriptional and translational levels, and has been widely studied in prokaryotes and eukaryotes (9–13). Interestingly, a previous study has revealed that transcription in archaea resembles that in eukaryotes and thus likewise is controlled by three core promoter elements located just upstream of the gene coding region, i.e., the TATA box, B recognition element (BRE), and transcriptional start site (TSS) (14). In archaeal methanogens, a consensus region has been identified in *Methanosarcina* and includes the 5 bp purine-rich BRE (CAAAA), the 8 bp TATA box (TTTATATA) that follows 2 bp downstream, and the pyrimidine–purine motif marking the TSS about 23 bp downstream from the TATA box (15, 16). Another important controlling factor of gene expression is the 5'-untranslated region (5'UTR), i.e., the sequence between the TSS and the start codon that is involved with posttranscriptional mRNA regulation (17). While some archaea [e.g., *Haloarchaea* (18)] can initiate transcription with a short 5′UTR, TSS mapping analysis has revealed that transcription initiation in the *Methanosarcina* species occurs with a rather long 100–500 bp 5′UTR (19), making the prediction of the promoter region in methanogens problematic. Recently, a promoter annotator dedicated to archaea has been developed (20), though the annotation of promoters for essential genes in *Methanosarcina* still remains effectively low and incomplete.

Thus far, only a few promoters and RBSs have been identified and characterized in *Methanosarcina* (Table 1), and these limitations are noticeable. For instance, there are no standard reporter proteins and methods for assessing promoter strengths and, in this context, will hinder making any comparisons. For the libraries consisting of mutational promoters, e.g., derivatives of the *minimal P_mcrB_* (a simplified version of the *mcrB* promoter containing only the core region) that were created by site mutation (15), the presence of slight sequence modifications can lead to unexpected recombination in expression cassettes. Finally, since the strengths of just a few wild-type promoters from *Methanosarcina* have been assessed, there is a limited understanding of the cellular protein expression patterns, particularly under different growth conditions. By comparison, a number of promoter–RBS combinations developed for gene expression in another model methanogen (i.e., *Methanococcus maripaludis*) have been reported (21–23). Given that these constructs include both wild-type and mutational combinations, this makes them rather useful genetic tools for heterologous protein expression in that organism.

**TABLE 1:** Previously reported promoters^*a*^ for gene expression in *Methanosarcina*

Promoter	Description	Reference
*mcr* promoter and derivatives	Promoter activities of the *mcr* operon and the mutational derivatives from *M. acetivorans* were assessed using β-galactosidase (lacZ) as the reporter	(15)
*atpH* promoter	Promoter activity of the *atp* operon from *M. acetivorans* was assessed using lacZ as the reporter	
*serC* promoters	Promoters of phosphoserine aminotransferase (*P_serC_; P_orf2_*) from *M. barkeri* were used for expressing *Escherichia coli proC* gene and Himar1 transposase gene (*tnp*) in *M. acetivorans*	(24)
*mcrB* promoter	Promoter of the *mcr* operon from *M. barkeri* was used for β-glucuronidase (UidA) expression in *M. acetivorans*	(25)
*mtaCB* promoters	Promoter activities of *mtaCB1/mtaCB2/mtaCB3* operons from *M. acetivorans* were assessed using UidA as the reporter	(26, 27)
Hydrogenase promoters	Promoter activities from hydrogenases in *Methanosarcina barkeri* (*ech, frh, vht, vhx,* and *hyp*) and *M. acetivorans* (*frh, vht, vhx,* and *hyp*) were assessed using UidA as the reporter	(28)
*hdr* promoters	Promoter activities from heterodisulﬁde reductase (*hdr*) operons in *M. acetivorans* were assessed using UidA as the reporter	(29)
*mreA* promoter	Promoter activity of the hypothetical protein (*mreA*) from *M. acetivorans* was assessed using UidA as the reporter	(30)
*cdhA* promoters	Promoter activities of *cdh* operons from *M. acetivorans* were assessed using UidA as the reporter	(31)

^*a*^ Promoters here indicate promoter–RBS combinations.

In this study, we constructed a promoter–RBS library to fine-tune gene expression levels in *M. acetivorans*. The library consists of wild-type promoter–RBS combinations from different methanogenic hosts as well as mutational variants developed by promoter–RBS hybrid and 5'UTR-engineering strategies. To determine the stability of these promoter–RBS combinations, their expression strengths were assessed under different growth conditions, in which either methanol (MeOH) or trimethyl amine (TMA) was present as the sole carbon and energy source. In addition, we also examined the influence of the growth phase on the gene expression from all promoter–RBS combinations.

## RESULTS

### Screening wild-type promoter–RBS combinations from different methanogens

**Fig 1:**
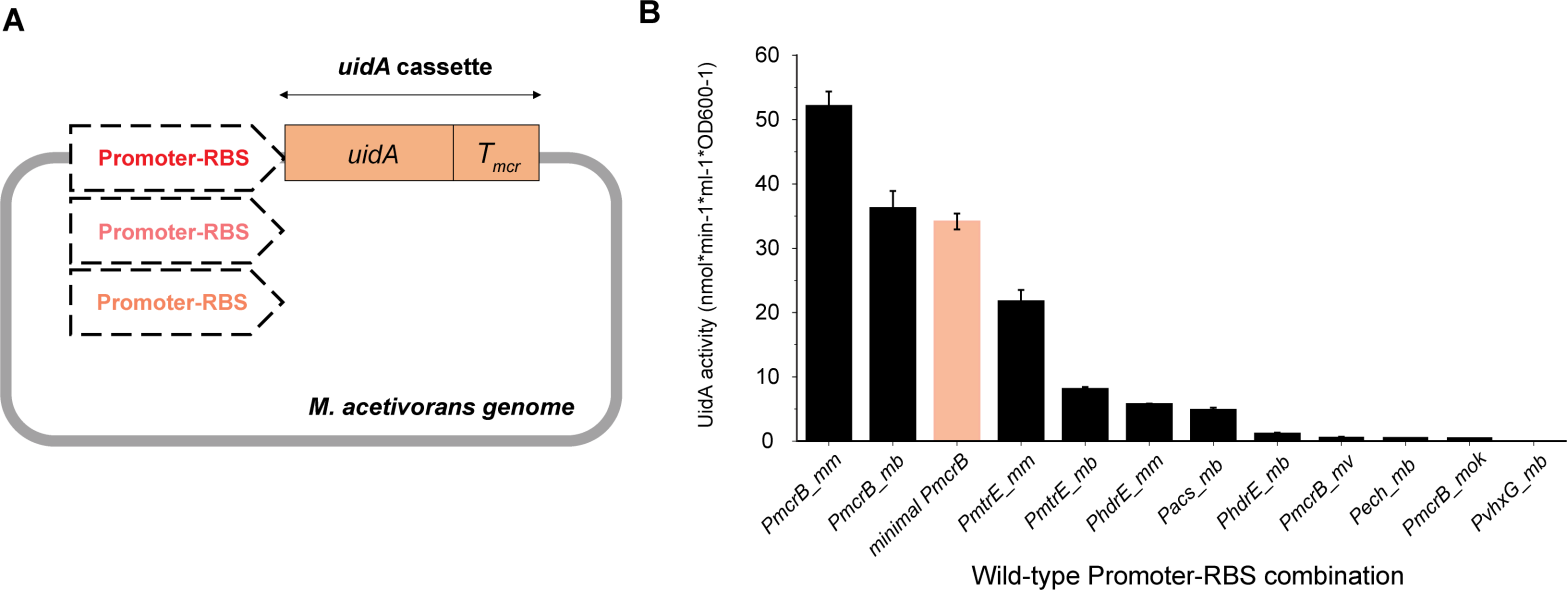
Characterization of wild-type promoter–RBS combinations from different methanogens. (**A**) Scheme for integration of the promoter–RBS*–uidA* cassette fusion into the *M. acetivorans* genome. The *uidA* cassette comprises the *uidA* gene from *E. coli* BL21 and the *mcr* terminator (*T_mcr_*) from *M. barkeri*. (**B**) Expression strength of the eleven wild-type promoter–RBS combinations (black bar) during growth on MeOH. The *minimal P_mcrB_* promoter (orange bar) was used as a control for performing strength comparison. Error bars represent the standard deviation of three biological replicates.

Eleven wild-type promoter–RBS combinations were selected from essential operons that regulate host energy metabolism (Table 2), aside from *P_vhxG_mb_*, which was previously reported as silent (28) and thus served as a negative control for screening. To avoid the instability caused by the possible recombination of highly similar sequences, candidate promoters were chosen from methanogenic species other than *M. acetivorans*. Also, considering the existence of long 5'UTRs, selected candidate promoters covered a 300–500 bp stretch of sequence (see Table S1), which precedes the start codon, with this depending on the distance of the neighboring upstream gene or operon. These sequences were amplified with the primers listed in Table S2. β-glucuronidase (UidA) from *E. coli* served as the reporter protein, and the *uidA* cassette was amplified from the plasmid used in a previous study (8) to construct the promoter–RBS*–uidA* cassette fusion (Fig. 1A). To obtain reliable gene expression data, fusion constructs were integrated into the *M. acetivorans* genome via the ΦC31 integrase-mediated site-specific recombination system (6). Strains carrying different promoter–RBS combinations were cultivated to the exponential phase (OD600 = 0.35–0.75) with MeOH as the sole carbon source. The corresponding expression strengths were assessed by using the UidA enzyme assay described in Materials and Methods. The *M. acetivorans* strain carrying the commonly used strong promoter *minimal P_mcrB_* (6, 15) acted as the reference for expression strength comparison.

**TABLE 2:** Wild-type promoter–RBS combinations used in this study

Name	Gene function	Organism
*P_mcrB_mm_*	Methyl-coenzyme M reductase (*mcrBDCGA*, MM_1244-MM1240)	*M. mazei* GÖ1
*P_mcrB_mb_*	Methyl-coenzyme M reductase (*mcrBCDGA*, Mbar_A0897-Mbar_A0893)	*M. barkeri* Fusaro
*P_mcrB_mok_*	Methyl-coenzyme M reductase (*mcrBDCGA*, Metok_0960- Metok_0956)	*Methanothermococcus okinawensis*
*P_mcrB_mv_*	Methyl-coenzyme M reductase (*mcrBDCGA*, Mvol_1124-Mvol_1126)	*Methanococcus voltae* A3
*P_mtrE_mm_*	Methyl-H_4_MPT methyltransferase (*mtrEDCBAFGH*, MM_1547 MM_1540)	*M. mazei* GÖ1
*P_mtrE_mb_*	Methyl-H_4_MPT methyltransferase (*mtrEDCBAFGH*, Mbar_A1262-Mbar_A1255)	*M. barkeri* Fusaro
*P_hdrE_mm_*	Heterodisulfide reductase (*hdrED*, MM_1843 MM_1844)	*M. mazei* GÖ1
*P_hdrE_mb_*	Heterodisulfide reductase (*hdrED*, Mbar_A1598-Mbar_A1599)	*M. barkeri* Fusaro
*P_acs_mb_*	CO dehydrogenase/acetyl-CoA synthase (*cdh*, Mbar_A0204-Mbar_A0199)	*M. barkeri* Fusaro
*P_ech_mb_*	Ferredoxin-dependent hydrogenase (*echABCDEF*, Mbar_A0152- Mbar_A0147)	*M. barkeri* Fusaro
*P_vhxG_mb_*	Methanophenazine-reducing hydrogenase (*vhxGAC*, Mbar_A1841- Mbar_A1839)	*M. barkeri* Fusaro

Among the eleven promoter–RBS combinations, *P_mcrB_mm_* and *P_mcrB_mb_* are both very strong (Fig. 1B). In this study, *P_mcrB_mm_* from *M. mazei* showed 150% greater strength than the *minimal P_mcrB_*, while the *P_mcrB_mb_* from *M. barkeri* was comparable in strength to the reference promoter. Strains carrying the other nine constructs yielded a lower expression, as their strengths were 60% below that of the *minimal P_mcrB_*. On the other hand, *P_mcrB_mok_* and *P_mcrB_mv_* from *Methanothermococcus* and *Methanococcus*, respectively, showed only 2% of the strength of the reference promoter, despite originating from strongly expressed *mcr* operons. *P_ech_mb_* and *P_vhxG_mb_* from *M. barkeri* were measured as the weakest promoters, showing less than 2% of the strength of the reference promoter and no strength, respectively. To better categorize the promoter–RBS combinations, each was assigned as either strong (> 100%), medium (20%–100%), or weak (< 20%) relative to the expression strength of the *minimal P_mcrB_*.

### Expanding the repertoire of medium-strength promoter–RBS combinations

Although the wild-type promoter–RBSs exhibit a broad range of strengths, their use for fine-tuning the gene expression is inadequate due to a lack of combinations with medium strengths. To expand the repertoire of medium-strength combinations (i.e., strengths between *minimal P_mcrB_* and *P_mtrE_mb_*), two strategies were implemented: (1) exchanging the promoter/RBS of the combinations to create hybrid variants and (2) developing additional wild-type combinations based on the results of a previous transcriptomic analysis of *M. acetivorans* (32).

**Fig 2:**
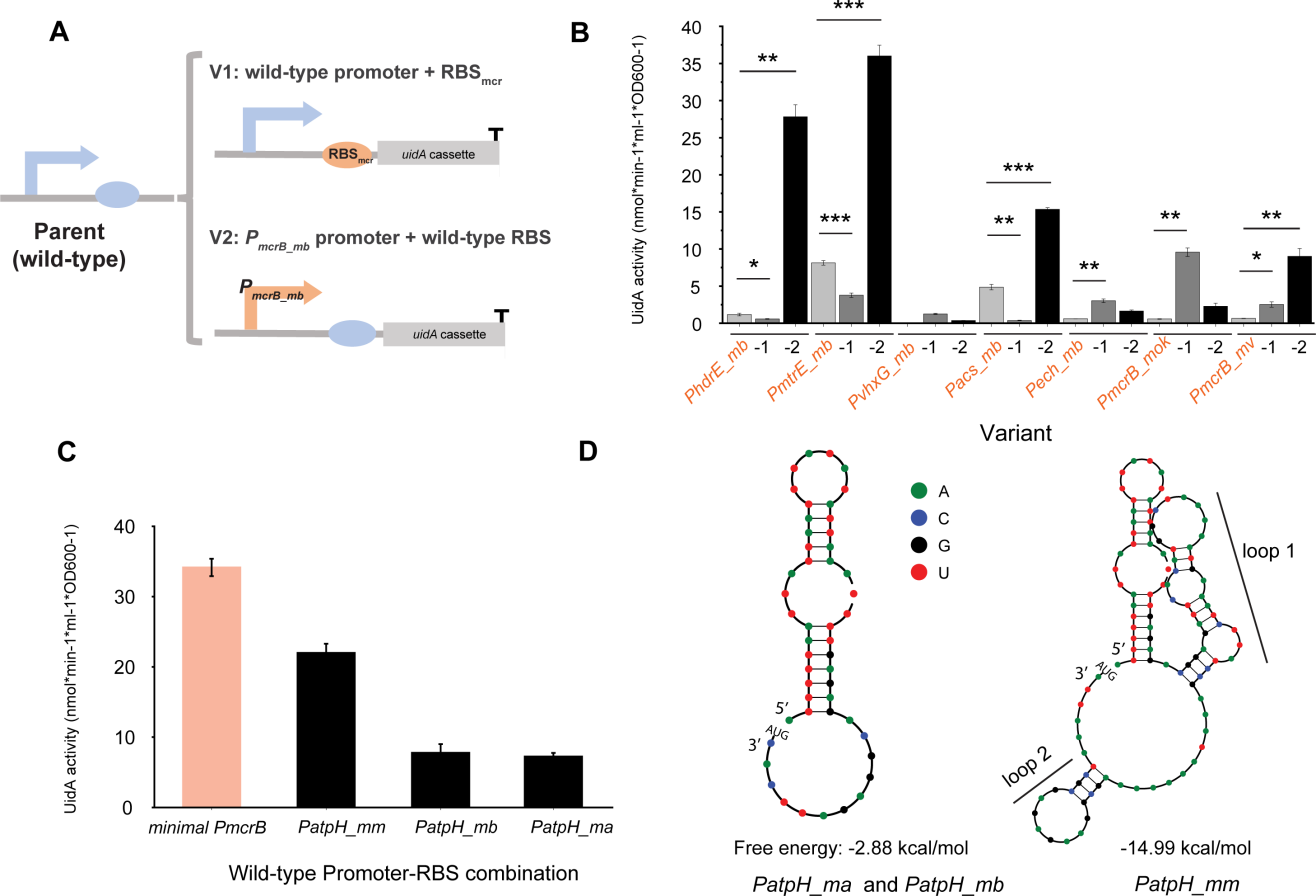
Strategies used to obtain medium-strength promoter–RBS combinations. Strategy 1. (**A**) Construction of promoter–RBS/hybrid variants (see the main text for details). Two variants (V1 and V2) are derived from a weak wild-type promoter–RBS combination (in blue). V1 possesses the weak wild-type promoter and the strong RBS_mcr_ from *P_mcrB_mb_*. V2 possesses the strong *P_mcrB_mb_* promoter and the weak wild-type RBS. *P_mcrB_mb_* is the strong wild-type promoter–RBS combination from *M. barkeri*. (**B**) Expression strength comparison of seven wild-type promoter–RBS combinations (orange font and light gray bar) and their promoter–RBS/hybrid variants. Variants of V1 (dark gray bar) and V2 (black bar) are denoted as −1 and −2, respectively. Error bars represent the standard deviation of three biological replicates (*P < 0.05, **P < 0.01, and ***P < 0.001). Strategy 2. (**C**) Expression strength of the wild-type *P_atpH_* promoter–RBS combinations from the *Methanosarcina* species. Promoters are derived as follows: *P_atpH_mm_* from *M. mazei*, *P_atpH_mb_* from *M. barkeri*, and *P_atpH_ma_* from *M. acetivorans*. The *minimal P_mcrB_* (orange bar) promoter was used for making expression strength comparison. (**D**) Predicted (partial) 5'UTR secondary structure of *P_atpH_ma_*, *P_atpH_mb_* (left), and *P_atpH_mm_* (right). Secondary structures and their free energies were predicted with NUPACK (https://www.nupack.org/) using default settings. Loops 1 and 2 are the predicted secondary structures in the extra 57-bp sequence of the 5'UTR in *P_atpH_mm_*. This additional sequence is not present in the 5'UTR of *P_atpH_ma_* and *P_atpH_mb_*. The start codon (AUG) in the mRNA sequence is indicated.

Low-strength promoter–RBS combinations might arise from a poor efficiency in either the promoter-driven transcription machinery or the translation step that depends on RBS strength (15). On this basis, two approaches were adopted to create hybrid variants of the seven weak-strength promoter–RBS combinations, i.e., *P_hdrE_mb_*, *P_mtrE_mb,_ P_vhxG_mb,_ P_acs_mb,_ P_ech_mb,_ P_mcrB_mok_*, and *P_mcrB_mv_*. Here, their expression strengths would be improved by (1) exchanging their wild-type RBS with the strong RBS_mcr_ of *P_mcrB_mb_* and (2) exchanging their wild-type promoter region with the strong *P_mcrB_mb_* promoter (i.e., V1 and V2 in Fig. 2A). It is worth noting that the RBS sequences encompass the 30-bp segments upstream of the start codon, which includes the putative RBS motif and the 4-bp ribosome standby site upstream of the RBS motif (15, 33). The corresponding sequences are marked in Table S1. Fourteen hybrid variants were constructed, and their expression strengths were characterized using growth conditions containing MeOH as the substrate (Fig. 2B). Remarkably, the expression strengths of two variant constructs (*P_hdrE_mb_*-2 and *P_mtrE_mb_*-2) that contained the *P_mcrB_mb_* promoter and their own wild-type RBS were significantly improved, showing increases of 23-fold and fourfold, respectively. In contrast, three variant constructs (*P_hdrE_mb_*-1, *P_mtrE_mb_*-1, and *P_acs_mb_*-1) exhibited a much lower expression strength than their wild-type construct, despite being fitted with the strong RBS_mcr_. Interestingly, for two variant constructs (*P_vhxG_mb_*-1 and *P_vhxG_mb_*-2), which were derived from the wild-type *P_vhxG_mb_* and showed no prior expression strength, their strengths became detectable after being equipped with either the strong RBS_mcr_ or the *P_mcrB_* promoter. Since the wild-type *P_vhxG_mb_* exhibited no detectable strength, it underwent no further characterization in this study.

As an alternative way to obtain more medium-strength combinations, the screening for additional wild-type promoter–RBS combinations was performed by using the results from a previous transcriptome analysis of *M. acetivorans* under different carbon sources (32). According to the transcriptomics results, the *atp* operon (formerly the *aha* operon) encoding the archaeal AOA1 ATP synthase complex in *M. acetivorans* is highly expressed in the presence of MeOH and acetate. In a previous study (15), gene expression from the *atpH* promoter in *M. acetivorans* was assessed using the LacZ reporter system and represented an ideal medium-strength promoter candidate. Hence, we constructed promoter–RBS combinations using the *P_atpH_ma_* from *M. acetivorans*, as well as its counterparts from *M. mazei* and *M. barkeri* (*P_atpH_mm_* and *P_atpH_mb_*, respectively), and then measured their expression strengths via the UidA enzyme assay. In this study, the results showed that these promoter–RBS combinations have medium-strength expression. The strength of *P_atpH_mm_* was threefold higher than that of *P_atpH_ma_*, whereas the *P_atpH_mb_* and *P_atpH_ma_* strengths were comparable to each other (Fig. 2C). To correlate these findings with any sequence similarities or differences between the three promoter–RBS combinations, we performed a MultAlin multiple alignment (see Materials and Methods). The results from the sequence alignment revealed the conserved regions for the three canonical core promoter elements (i.e., BRE, TATA box, and TSS) and a single RBS motif. Intriguingly, *P_atpH_mm_*, which was inferred to be much stronger than the other two promoters (*P* = 0.0008 in two-tailed *t*-test), possessed a long 57-bp stretch of sequence between the putative RBS and the start codon (Fig. S1). NUPACK (see Materials and Methods) predictions of the 5'UTR secondary structure revealed the formation of two loops (i.e., loops 1 and 2 in Fig. 2D) within this extra sequence in *P_atpH_mm_*. Since our aim was to select candidate promoters from methanogenic species other than *M. acetivorans*, we opted to construct promoter–RBS combinations using the *P_atpH_mm_* and *P_atpH_mb_* promoters. By using a two-pronged strategy (see above), we generated six additional medium-strength promoter–RBS combinations (i.e., *P_hdrE_mb_*-2, *P_acs_mb_*-2, *P_mcrB_mok_*-1*, P_mcrB_mv_*-2*, P_atpH_mm_,* and *P_atpH_mb_*) to expand the overall range and repertoire of strong–medium–weak combinations.

### Screening for high-strength variants by the 5'UTR-engineering of *P_mcrB_mm_*

**Fig 3:**
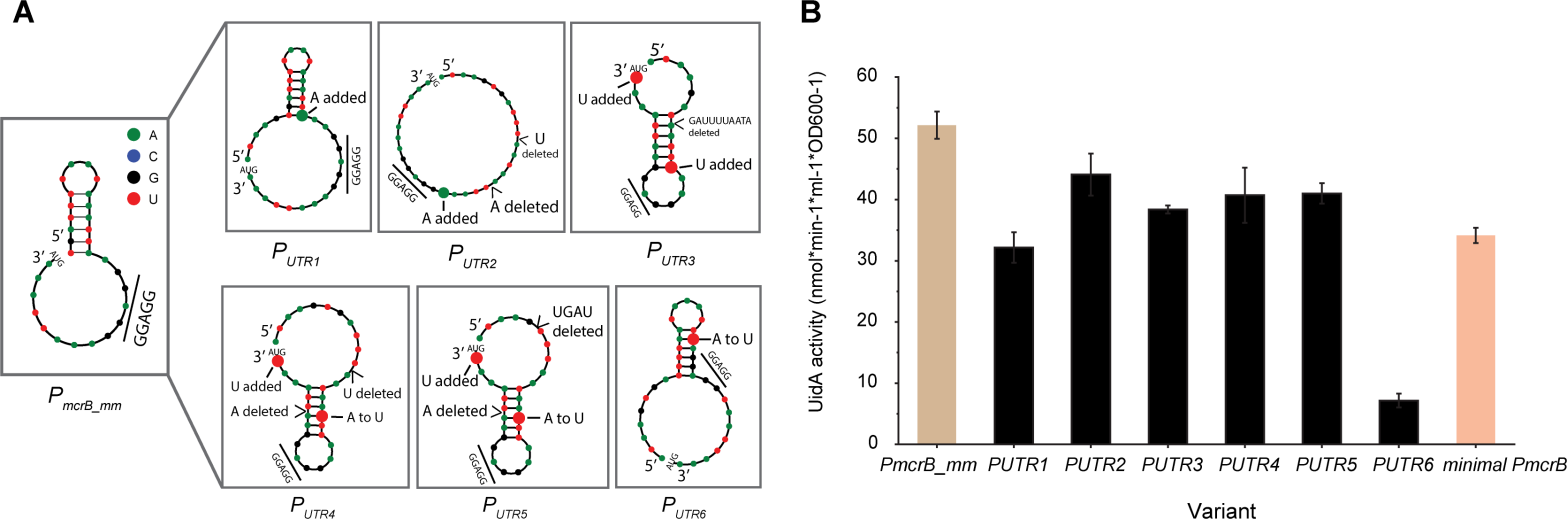
Engineered promoter–RBS combination variants with stronger expression than *P_mcrB_mm_*. (**A**) RNA secondary structure predictions of the last 30-bp sequence in the *P_mcrB_mm_* 5'UTR for six promoter–RBS combination variants (*P_UTR1_*, *P_UTR2_*, *P_UTR3_*, *P_UTR4_*, *P_UTR5_*, and *P_UTR6_*). Mutation sites in each variant are annotated in the secondary structure. RNA secondary structures were predicted with NUPACK using default settings. RNA secondary structures for the full-length 5'UTR sequence of the six promoter–RBS combination variants as well as their free energies are provided in Fig. S2. The consensus RBS motif sequence (5´-GGAGG) in the *Methanosarcina* species and the start codon (AUG) in the mRNA sequence are both indicated. (**B**) Expression strengths of the *P_mcrB_mm_* variant strains (black bar) during growth on MeOH. Strength comparisons included using wild-type *P_mcrB_mm_* (beige bar) and *minimal P_mcrB_* (orange bar) as controls. Error bars represent the standard deviation of three biological replicates.

Typically, a strong promoter–RBS combination is essential when high protein yields are required for biotechnological applications. In this study, there are only three promoter–RBS combinations (i.e., *P_mcrB_mm_*, *P_mcrB_mb_*, and *P_mtrE_mb_*-2) that yield stronger expression than the reference promoter, which limits the use of our promoter–RBS library for applications involving the overexpression of proteins. Previously, in a *Bacillus* species, engineering the 5′UTR of a strong constitutive promoter led to an even stronger promoter and greatly increased protein production (34). Inspired by this strategy, we attempted to create mutational variants by engineering the 5'UTR of our strongest promoter (*P_mcrB_mm_*) and thereby provide promoter candidates with even greater expression strength. Based on the predicted free energy and the RNA secondary structure of *P_mcrB_mm_*, a total of six rationally designed variants were created: five (*P_UTR1_*, *P_UTR2_*_,_*P_UTR3,_ P_UTR4_,* and *P_UTR5_*) containing various mutations within the last 30 bp of *P_mcrB_mm_* and one (*P_UTR6_*) containing the consensus RBS (5´-TTAGGAGGT) sequence of *Methanosarcina* (Fig. 3A; Fig. S2). The six variants were assessed for expression strength in the presence of MeOH, with *minimal P_mcrB_* serving as the reference for strength comparisons. All six *P_mcrB_mm_* variants produced statistically significant differences in strength (*P* < 0.05 in two-tailed *t*-test) when compared to the reference promoter. Here, the *P_UTR2_*, *P_UTR3_*, and *P_UTR5_* variants were 1.5-fold stronger than the reference (*P* < 0.05 in two-tailed *t*-test), whereas the *P_UTR6_* variant retained only 20% of the expression strength of the reference (Fig. 3B). In total, this strategy yielded four additional high-strength promoter–RBS combinations, though none exceeded the expression strength of the wild-type *P_mcrB_mm_* promoter.

### Strength characterization of the promoter–RBS library in the presence of MeOH and TMA

**Fig 4:**
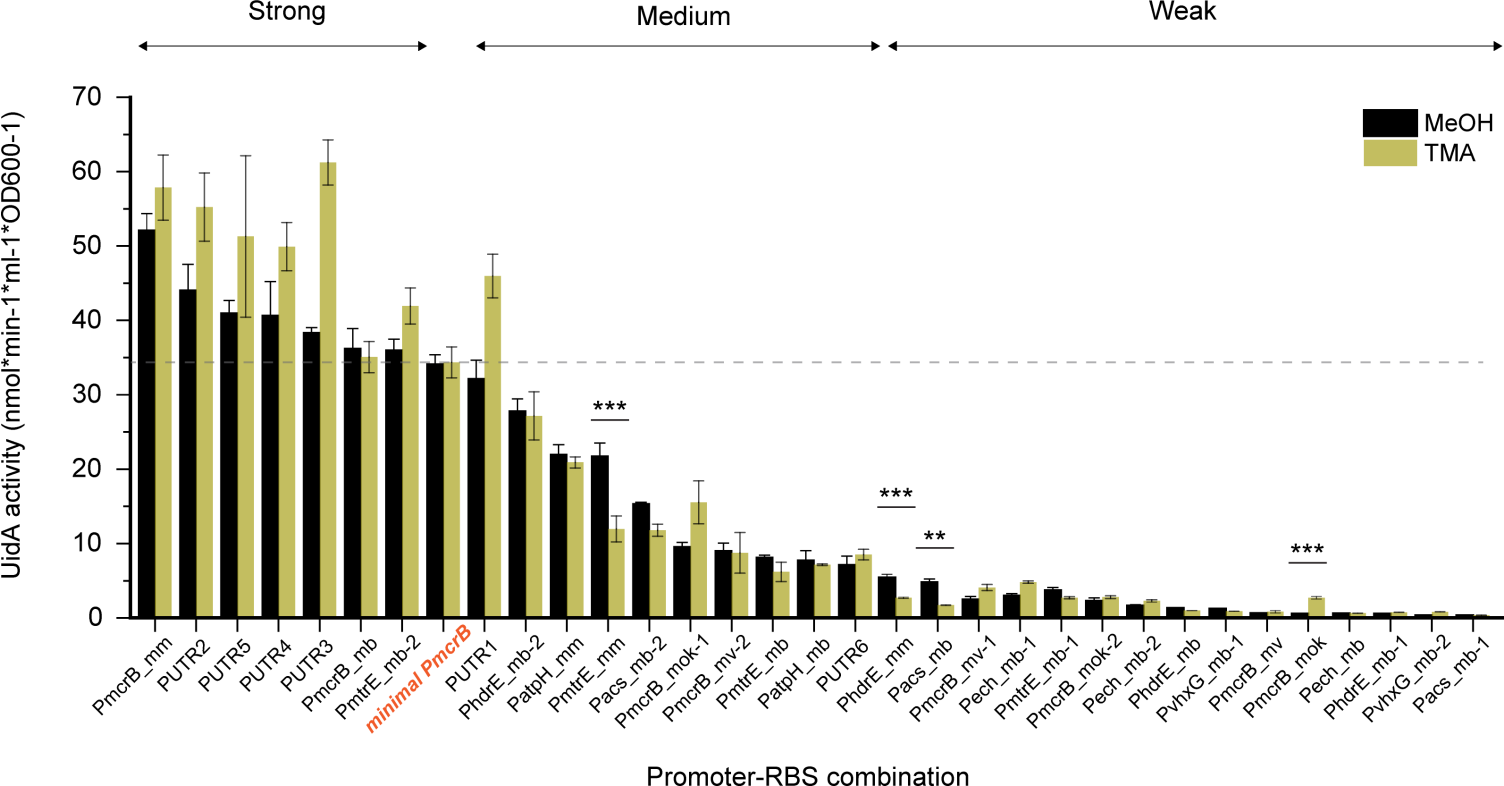
Expression strength of all 33 promoter–RBS combinations grown on MeOH or TMA as the sole carbon source. Growth on MeOH (black bar) or TMA (brass bar) is indicated. The *minimal P_mcrB_* promoter (orange font) was used as the reference to categorize the promoter–RBS combinations as either strong (> 100%), medium (20%–100%), or weak (< 20%) (dashed line). Error bars represent the standard deviation of three biological replicates (**P < 0.01 and ***P < 0.001).

Our study yielded a library of 33 different promoter–RBS combinations with a 140-fold range in expression strength in the presence of MeOH (Fig. 4). Based on comparisons with the reference *minimal P_mcrB_* promoter, each of these promoter–RBS combinations was assigned into one of three categories: strong, medium, or weak. Among these 33 combinations, eight were strong (with expression strengths of up to 150% of the reference), ten were medium (with expression strengths ranging between 20% and 100% of the reference), and fifteen were weak (with expression strengths less than 20% of the reference). To determine whether the expression strengths of the various promoter–RBS combinations remain similar in other methylated substrates, measurements were carried out in the presence of TMA (MeOH-grown *M. acetivorans* cells were adapted to TMA for at least 20 generations prior to taking measurements). Over half of the promoter–RBS combinations shared comparable expression strengths in both growth substrates, whereas 13 of them were found to be stronger in the presence of TMA. This was particularly evident for the *P_mcrB_mok_* combination, whose expression strength increased over fivefold in TMA (*P* < 0.001 in two-tailed *t*-test). On the other hand, the *P_mtrE_mm_*, *P_hdrE_mm_*, and *P_acs_mb_* combinations showed at least a twofold decrease in expression strength in TMA.

### Growth phase effect on the gene expression of the promoter–RBS combinations

To assess the effect of the 33 promoter–RBS combinations on growth of *M. acetivorans* cells, growth curves of the corresponding strains were determined and compared, with an empty vector strain (i.e., PZ0 strain containing the *uidA* cassette but lacking the promoter–RBS sequence) serving as the experimental control (Fig. S3). No significant difference in cell growth was observed among the various “promoter–RBS combination” strains, as all respective cultures reached an OD600 >1.7 after a 40-hour incubation period (with an OD600 = 1.77 ± .03 for the experimental control). This indicates that none of the promoter–RBS combinations is detrimental to cell fitness, thus allowing them to be used for gene expression purposes in *M. acetivorans*.

**Fig 5:**
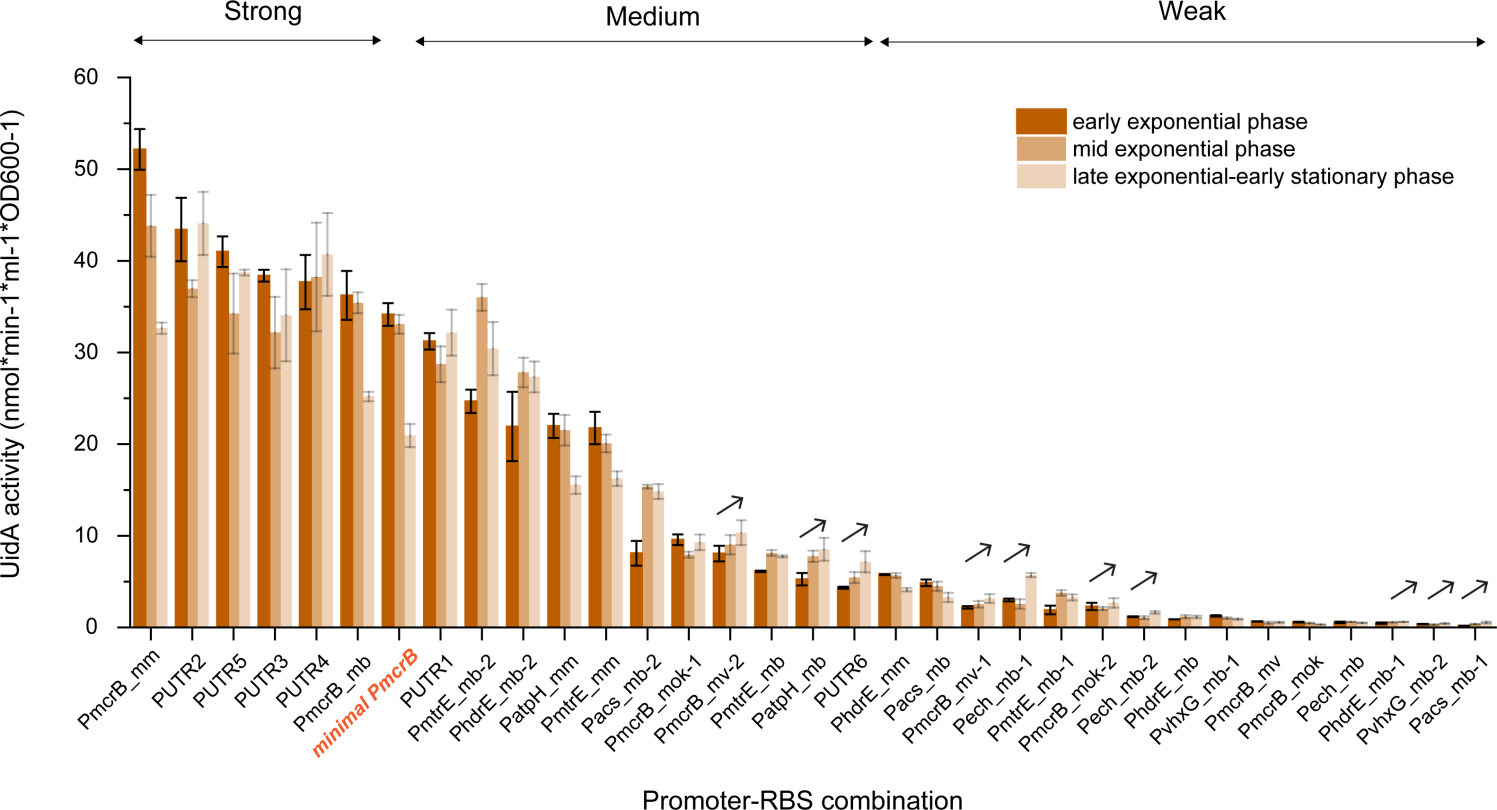
Expression strength of all 33 promoter–RBS combinations during different growth phases. MeOH was used as the sole carbon and energy source. Phases of cell growth are marked as follows: early exponential phase (OD600 = 0.3–0.5) (dark orange bar), mid-exponential phase (OD600 = 0.5–0.8) (orange bar), and late exponential–early stationary phase (OD600 = 0.8–1.2) (light orange bar). Promoter–RBS combinations showing increased gene expression and cell growth are indicated by black arrows. Error bars represent the standard deviation of three biological replicates.

In a previous study, it was determined that promoter–RBS-mediated gene expression is a dynamic balancing of RNA polymerase and ribosome availability in growing cells (35). Thus, to investigate the stability of our promoter–RBS combinations, the gene expression of the corresponding strains was assessed during various phases of cell growth, i.e., the early exponential (OD600 = 0.3–0.5), mid-exponential (OD600 = 0.5–0.8), and late-to-early stationary phases (OD600 = 0.8–1.2). Among the 33 promoter–RBS combinations, 23 of them showed the highest gene expression during either the early exponential or mid-exponential phases, whereas ten of them showed an increased gene expression during the early stationary phase (Fig. 5). Additionally, a correlation was also evident between expression strength and growth phase, as the majority of high-strength combinations showed their highest gene expression at the early exponential phase, though most weak-strength combinations showed their maximum gene expression during the stationary phase.

## DISCUSSION

In this study, we constructed a library of 33 promoter–RBS combinations to fine-tune gene expression in *M. acetivorans* over a 140-fold spectrum. The wild-type promoter–RBS combinations in our library were derived from methanogens other than *M. acetivorans*, which lowers the risk of any unexpected homologous recombination, particularly in situations when a wide range of promoter–RBS combinations is needed, such as for fine-tuning essential gene expression and balancing metabolic fluxes.

Our findings revealed that the wild-type promoter–RBS combinations from phyletically distant methanogen taxa result in lower (weak) gene expression in *M. acetivorans*. The *P_mcrB_oki_* and *P_mcrB_mv_* combinations from more distant *Methanococcus* and *Methanothermococcus*, respectively, exhibited only 2% of the expression strength of the reference promoter (*minimal P_mcrB_*) combination. This contrasts with the *P_mcrB_mm_* and *P_mcrB_mb_* combinations from closely related *M. mazei* and *M. barkeri*, respectively, which drive much stronger gene expression. Such differences can be attributed to alterations in the transcription and translation factors of these methanogens. Such regulatory factors recognize the TATA box, BRE, and RBS motif, collectively the essential elements for initiating transcriptional and translational processes (36). Although only a few methanogen-specific transcription factors have been characterized, the observation that the promoter and RBS sequences of the weak and strong wild-type combinations (Table S1) have <50% similarity suggested that transcriptional regulation can differ between methanogenic orders with or without the cytochrome. Differences in energy conservation and substrate metabolism (27, 37–39) might trigger the activation of regulatory signals in gene expression that also affect the expression strength. The *P_ech_mb_* and *P_vhxG_mb_* combinations from the hydrogenases of *M. barkeri* barely evoked any detectable gene expression in our study, which is consistent with previous study results (28). Additionally, three (strong, medium, and weak) expression levels were empirically defined in this study to facilitate a better comparison between the strengths of the promoter–RBS combinations. Promoter–RBS combinations were defined as “strong” based on their expression strengths relative to the *minimal P_mcrB_* promoter, which is commonly regarded as being strong in *M. acetivorans*. Promoter–RBS combinations defined as either “medium” or “weak” were determined based on all expression strengths not categorized as strong. While in a previous study the *serC* promoter from *M. barkeri* Fusaro was categorized as “medium-strength” during gene expression in *M. acetivorans* (24), this did not apply to our work since the strength of the *serC* promoter is <1% of that for the wild-type *mcrB* promoter, and in our case, the majority of promoter–RBS combinations demonstrated much higher expression levels. For the promoter–RBS combination *P_hdrE_mb_* derived from *M. barkeri*, which regulates the gene expression of essential heterodisulfide reductase (HdrED1), its expression strength in *M. acetivorans* was fourfold lower than the *P_hdrE_mm_* version from *M. mazei*, irrespective of the phylogenetic similarities. To rationalize this finding, we aligned the DNA sequence of their promoter–RBS regions (*P_hdrE_mm_*, *P_hdrE_mb,_* and *P_hdrE_ma_*) as well as predicted their RNA secondary structures (Fig. S4). Expectedly, their sequences shared a common set of core promoter (BRE, TATA box, and TSS) and RBS elements. Thus, the lower expression strengths we observed might arise from upstream- and/or downstream-induced transcriptional regulation and/or the possibility of RBS hairpin formation affecting translation by disrupting ribosomal binding. Since the expression strength of the *P_hdrE_mm_* combination strain decreased twofold (*P* < 0.001 in two-tailed *t*-test) after the switch in growth substrates from MeOH to TMA, an effect on what regulates transcription might be a reasonable explanation. Similarly, the expression strength of wild-type *P_hdrE_ma_* from *M. acetivorans* had previously seen a fourfold increase during the same transition in growth substrates (29), though this change was not evident in our study. As for the apparent discrepancy in expression strengths, this might depend on the different lengths of the sequence used for constructing the promoter–RBS combinations, i.e., 1,000 bp in the previous study and 500 bp in our present study. It is our speculation that the additional upstream sequence possibly holds a regulatory region that becomes active in the absence or presence of certain growth substrates. The variability in expression strength among the three promoter–RBS combinations (*P_hdrE_mm_*, *P_hdrE_mb_*, and *P_hdrE_ma_*) indicates that substrate-dependent transcriptional regulation has a role in *M. mazei*, *M. barkeri*, and *M. acetivorans*, particularly since transcriptional regulation has already been reported in other methanogens (16, 30–32, 40).

Since transcriptional and translational activities are controlled by the promoter and RBS, respectively, their exchange and/or modification to create “hybrids” has the potential to greatly affect the initiation rates of the wild-type promoter–RBS combinations and thereby the overall gene expression strength (41–43). To broaden the range of medium-strength combinations, an attempt was made to augment the strength of weak promoters by exchanging their RBSs with the sequence of the relatively stronger RBS_mcr_ motif. Unexpectedly, some of these promoter–RBS hybrid-derivatives (*P_hdrE_mb_*-1, *P_mtrE_mb_*-1, and *P_acs_mb_*-1) had exhibited even lower expression strengths than the wild-type combinations. This suggests that the original weakness of the wild-type promoter–RBS combinations is related to posttranscriptional regulation via mRNA structures [e.g., attenuators and riboswitches (44)] instead of simply to the RBS strength. This hypothesis appears to be valid since the hybrid combination variants (*P_hdrE_mb_*-2 and *P_mtrE_mb_*-2) equipped with their own wild-type RBS but an identical *P_mcrB_mb_* promoter sequence (see Fig. 2) had displayed increased expression strengths of over twenty-fold and fourfold, respectively. The wild-type *P_vhxG_mb_* promoter–RBS combination had no measurable expression, and its putative RBS motif was not discernible since it lacked sufficient similarity with the consensus *Methanosarcina* RBS sequence (5′-GGAGG). Therefore, we decided to exclude it from Table S1. Interestingly, a weak expression strength became detectable in the *P_vhxG_mb_* promoter–RBS combination after we replaced its own RBS with the strong *RBS_mcr_*, causing the variant *P_vhxG_mb_* −1 and wild-type *P_hdrE_mb_* combinations to be comparable in strength. This appears to indicate that the RBS sequence of wild-type *P_vhxG_mb_* might either be disrupted or have a very low binding affinity for ribosomes. Moreover, the variant *P_vhxG_mb_*-2 created using the V2 hybrid strategy had gained a trace amount of expression strength instead of none. This supports the notion that the wild-type RBS might be functional with an extremely low translation efficiency, making the expression strength essentially undetectable when paired with a weak promoter. While the putative RBS sequence of the wild-type *P_ech_mb_* (5′-ATCGGAGGA) shares more than 80% identity with the counterpart (5′-ATAGGAGGA, which was shown to be strong via the V2 hybrid strategy) in the wild-type *P_hdrE_mb_* combination, a previous study found that double site-mutations (TT to GA) in the minimal *mcrB* RBS sequence can cause a 50% loss in expression strength in *M. acetivorans* (15), suggesting that mutations can significantly affect the RBS strength.

Modification of the 5′UTR by rational design for gene expression engineering was previously proven effective in *Bacillus* (34). However, attempting the same with our strongest *P_mcrB_mm_* promoter had failed to produce any promoter–RBS combinations with even higher expression strengths. Still, influencing the availability of the RBS to increase ribosomal binding to mRNA has been shown to be an effective approach for improving translation efficiency (45, 46). In our study, the RBS-mutated *P_UTR6_* combination variant showed a significant decrease in expression strength. Based on its predicted RNA secondary structure, some of the stem-loop appears to be part of the RBS sequence (Fig. S2), which then likely hinders ribosomal binding and affects the efficiency of translation. Modifying the upstream hairpin stem or the spacing downstream of the RBS (but not its sequence motif) had little effect on the expression strength of the variants, which remained strong. Given our lack of success to increase the expression strength via 5´UTR engineering, we speculate that a maximal protein synthesis rate in *M. acetivorans* might exist, as it balances the allocation of limited cellular resources between heterologous protein expression and cell growth (47). Here, it is not credible to conclude that the strongest *P_mcrB_mm_* combination-controlled gene expression has reached the maximal protein yield in *M. acetivorans*, simply based on a previous study that demonstrated that methyl-coenzyme M reductase (MCR) accounts for up to 7% of the total cellular protein in methanotrophic archaea (48).

We also tested the expression strength of all promoter–RBS combinations during the different phases of cell growth. Although we measured about 30% reduction in expression strength for the wild-type *mcr* promoter–RBS combinations (*P_mcrB_mm_* and *P_mcrB_mb_*) when cells entered the late-exponential phase, the strong *P_UTR_* combination variants (*P_UTR2_*, *P_UTR3_*, *P_UTR4_*, and *P_UTR5_*) derived from *P_mcrB_mm_* had maintained their expression strengths during all growth phases. These results suggest that the rational engineering of the promoter–RBS combinations had led to gene constructs with a stable and reliable expression.

To summarize our study, we constructed a promoter–RBS library of graded strength for fine-tuning gene expression in the two most used growth substrates (MeOH and TMA) for *M. acetivorans*. It must be pointed out that the expression strengths can vary depending on the nature of the protein being expressed, particularly if it has been shown to affect cell fitness or impose a metabolic burden on host cells. Consequently, the actual expression strengths of the promoter–RBS combinations might differ numerically if the protein of interest can exert a metabolic interference in *M. acetivorans* cells. Nevertheless, our constructed library is a valuable tool that enables the protein expression to be fine-tuned for physiological studies and intricate metabolic engineering in *M. acetivorans*.

## MATERIALS AND METHODS

### Strains and media

*E. coli* Top10 (Thermo Scientific) was used as the cloning host for plasmid construction. *E. coli* transformants were selected using lysogeny broth (LB) solid medium supplemented with 100 µg/mL ampicillin. *M. acetivorans* WWM73 was used as the host strain in this study, and its derivatives are listed in Table S3. High-salt (HS) medium supplemented with 2 µg/mL puromycin (InvivoGen, Inc.) and either 125 mM MeOH or 50 mM TMA was used for cultivating *M. acetivorans* in a single-cell form at 37°C (49). The HS solid medium containing 1.4% agar and 2 µg/mL puromycin was used for screening *M. acetivorans* transformants.

### Primers and plasmid construction

All primers and plasmids used in this study are listed in Table S2 and S3, respectively. PrimeSTAR Max DNA Polymerase (Takara Bio) was used to amplify gene fragments for plasmid construction. SapphireAmp Fast PCR Master Mix (Takara Bio) was used for colony PCR to verify the *E. coli* and *M. acetivorans* transformants. HiFi DNA Assembly Master Mix (New England BioLabs) was used for Gibson assembly cloning. The NEB Golden Gate Assembly Kit (BsaI-HFv2) (New England BioLabs) was used for assembling DNA fragments.

### DNA transformation

Polyethylene glycol (PEG) 4000-mediated transformation of *M. acetivorans* was performed with 1 µg DNA by following the method described previously (50). *M. acetivorans* transformants were grown on the HS solid medium and incubated in an anerobic jar with a controlled headspace of N_2_/CO_2_/1% H_2_S (75/20/5) at 37°C for 10–15 days. Chemically competent *E. coli* cells were used for the transformation of assembled plasmids.

### β-Glucuronidase enzyme assay

Activity measurements were carried out using a previously described assay method (21), but with slight modifications. Briefly, fresh *M. acetivorans* cultures were grown at 37°C in HS medium supplemented with 2 µg/mL puromycin and either 125 mM MeOH or 50 mM TMA until reaching an optimum cell density at OD600. Afterward, a 1-mL aliquot of the culture was collected using a clinical centrifuge (13,000 rpm for 5 minutes at room temperature) and assayed for β-glucuronidase activity as follows: (**a**) the culture supernatant is discarded, and the cell pellet is resuspended in a micro-centrifuge tube with a 400-µL aliquot of resuspension buffer consisting of 50 mM Na-phosphate buffer, pH 7.0, and protease inhibitor (100 x Halt Protease Inhibitor Cocktail, EDTA-free, Thermo Scientific) and incubated on ice with an open lid for 5 minutes; (**b**) the samples are then re-centrifuged (as above) to separate the cell debris from the cell-free extract (CFE), which is collected into another tube; (**c**) a 50-µL aliquot of CFE is diluted with 450 µL resuspension buffer, giving a 500 µL total volume (a 500-µL aliquot of resuspension buffer in a separate tube serves as the blank control); (**d**) experimental and control samples (with an open lid) are transferred to a benchtop thermocycler (Eppendorf ThermoMixer C), supplemented with a 40-µL aliquot of 10 mg/mL 4-nitrophenyl β-D-glucuronide (4 NPG) dissolved in the resuspension buffer and then incubated with shaking (350 rpm) for 15 minutes at 37°C; (**e**) the assay reaction is stopped with a 400-µL aliquot of sodium carbonate solution (200 mM Na_2_CO_3_); (**f**) reaction samples are diluted beforehand (optional) and measured for absorbance at OD405; and (**g**) the enzyme activity is calculated using a standard curve of *E. coli* β-glucuronidase (Roche) (see Fig. S5). One unit (U) of β-glucuronidase activity is defined as the amount of enzyme that catalyzes the conversion of 1 µmol of 4 NPG at 37°C and pH 7. All experiments were carried out in triplicate.

### Multiple sequence alignment and RNA secondary structure prediction

A multiple alignment of DNA sequences was created in MultAlin (http://multalin.toulouse.inra.fr/multalin/multalin.html) using default settings. RNA secondary structure predictions were made with the online version of NUPACK (https://www.nupack.org/) using default settings.

## Data Availability

The plasmid sequences can be found via the hyperlinks provided in Table S3.

## SUPPLEMENTAL MATERIAL

Supplemental material aem.01092-24-s0001.pdf Figures S1 to S5; Tables S1 to S3.

ASM does not own the copyrights to Supplemental Material that may be linked to, or accessed through, an article. The authors have granted ASM a non-exclusive, world-wide license to publish the Supplemental Material files. Please contact the corresponding author directly for reuse.
