# Supplementary material for: A promoter–RBS library for fine-tuning gene expression in Methanosarcina acetivorans
Source: Appl Environ Microbiol. 2024 Aug 12;90(9):e01092-24. doi: 10.1128/aem.01092-24 (PMC11409679; doi:10.1128/aem.01092-24)
Supplement: Supplemental material — Figures S1 to S5; Tables S1 to S3. [file aem.01092-24-s0001.pdf]

Supplementary material

A promoter-RBS library for fine-tuning gene expression in *Methanosarcina acetivorans*

Ping Zhu[a], Mariana Molina Resendiz[a], Ingemar von Ossowski[a], Silvan Scheller[a]

a. Department of Bioproducts and Biosystems, School of Chemical Engineering, Aalto University, Espoo, Finland

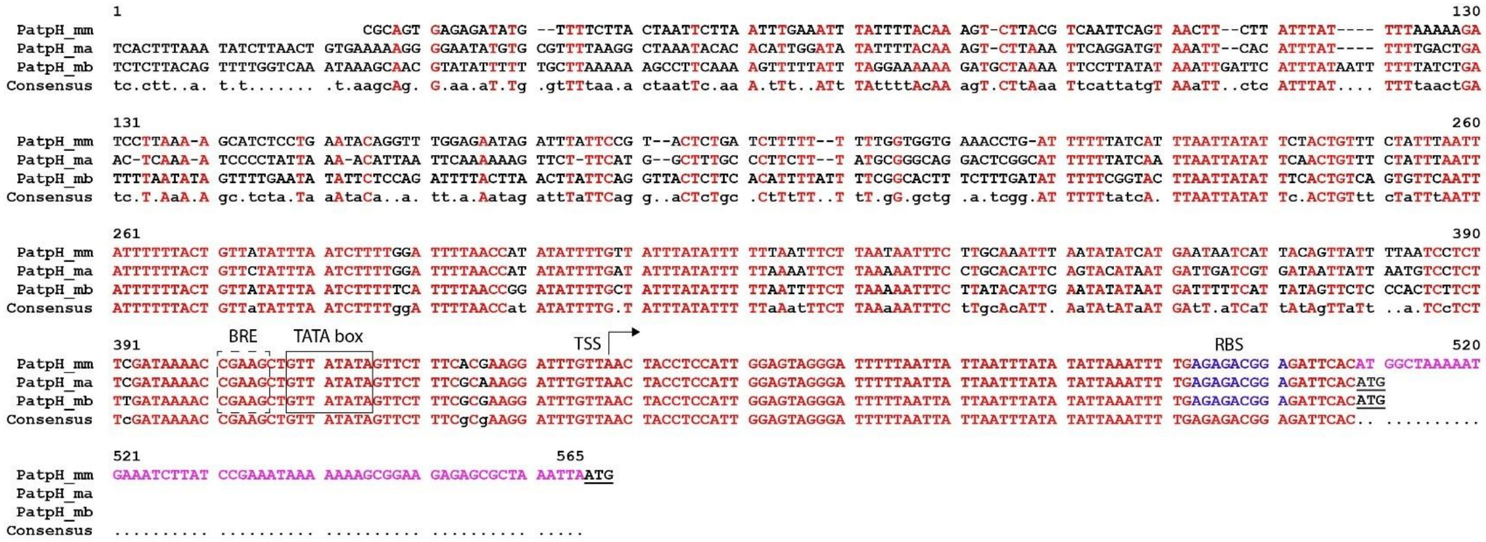

Figure S1. Sequence alignment of *aptH* promoter-RBS from different *Methanosarcina* species. Promoter sequences are derived as follows: P_{atpH_mm} from *M. mazei*, P_{atpH_mb} from *M. barkeri*, and P_{atpH_ma} from *M. acetivorans*. A multiple alignment of DNA sequences was performed with MultAlin (http://multalin.toulouse.inra.fr/multalin/multalin.html) using default settings. Highly conserved regions (> 90% similarity) are indicated (red font). The putative BRE (dashed line bordering), TATA box (solid line bordering), and TSS (black arrow) of P_{atpH_ma} were previously identified (1). The putative RBS sequence is shown (purple). An additional 57 bp sequence downstream of the putative RBS of P_{atpH_mm} is shown (pink). The start codon (ATG) is underlined in each DNA sequence.

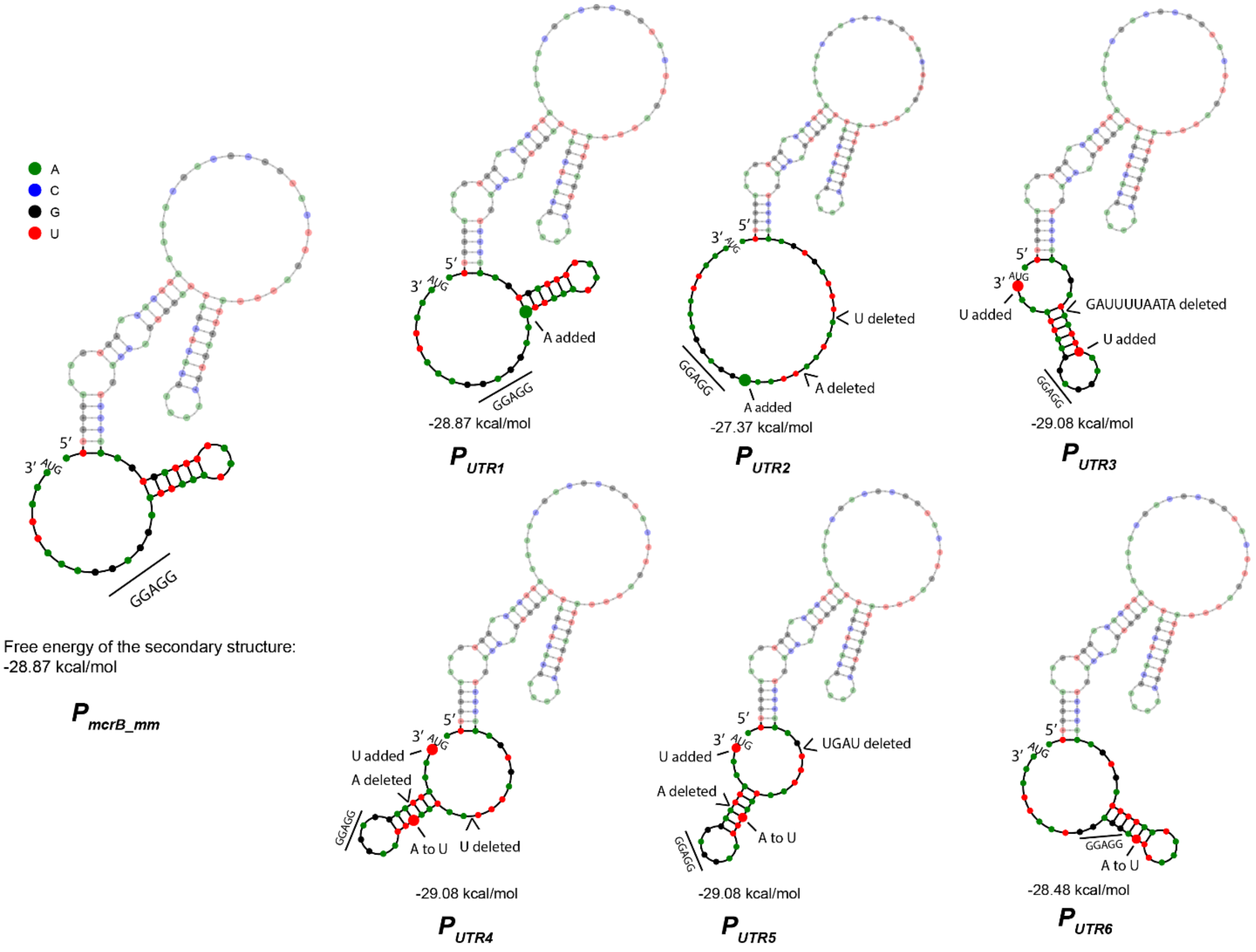

Figure S2. Predicted RNA secondary structures of the entire 5'UTR sequence in the promoter-RBS combination of *P_{mcrB_mm}* and its six variants. RNA secondary structures and their free energies were predicted with NUPACK (https://www.nupack.org/) using default settings. All 5'UTRs are in the 5'-to-3' direction. Bases in the RNA sequence are designated as colored dots (A, green; C, blue; G, black; and U, red). DNA sequences for the promoter (P_{mcrB_mm} from *M. mazei*) and consensus RBS motif (GGAGG) are underlined. Promoter-RBS combination variants (P_{UTR1}, P_{UTR2}, P_{UTR3}, P_{UTR4}, P_{UTR5}, and P_{UTR6}) are derived from P_{mcrB_mm}. Mutation sites in each variant are annotated in the secondary structure. The location of the start codon (AUG) is shown.

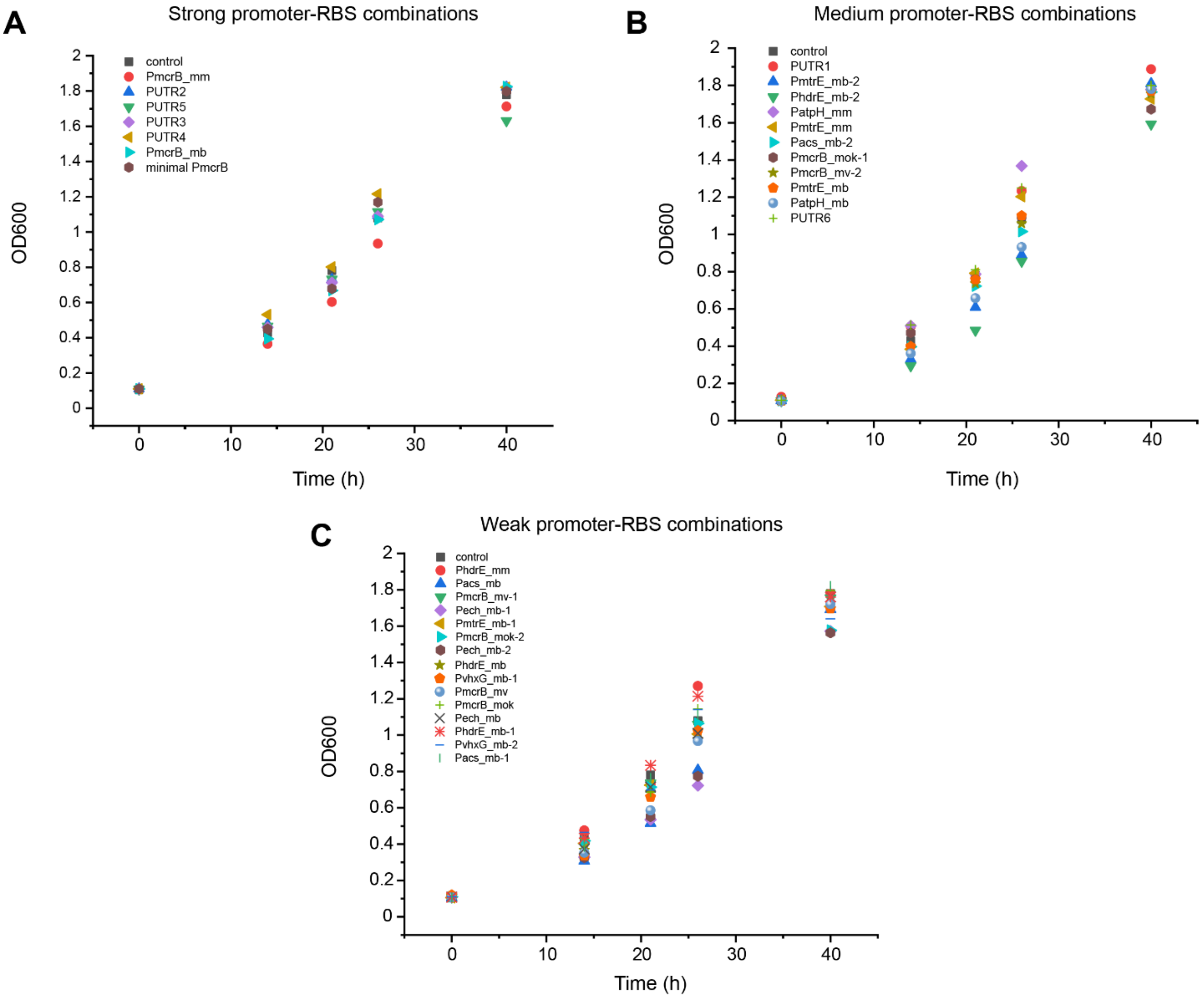

Figure S3. Growth curves of various promoter-RBS combination strains in MeOH. For comparative purposes, all data points represent an average value of three replicates. **(A)** Strains expressing strong promoter-RBS combinations. **(B)** Strains expressing medium promoter-RBS combinations. **(C)** Strains expressing weak promoter-RBS combinations. An empty vector strain (PZ0 strain containing the *uidA* cassette but lacking the promoter-RBS sequence) is the control (dark gray square). All cultures reached an OD600 > 1.7 after a 40-hour incubation period (OD600 = 1.77 for the control).

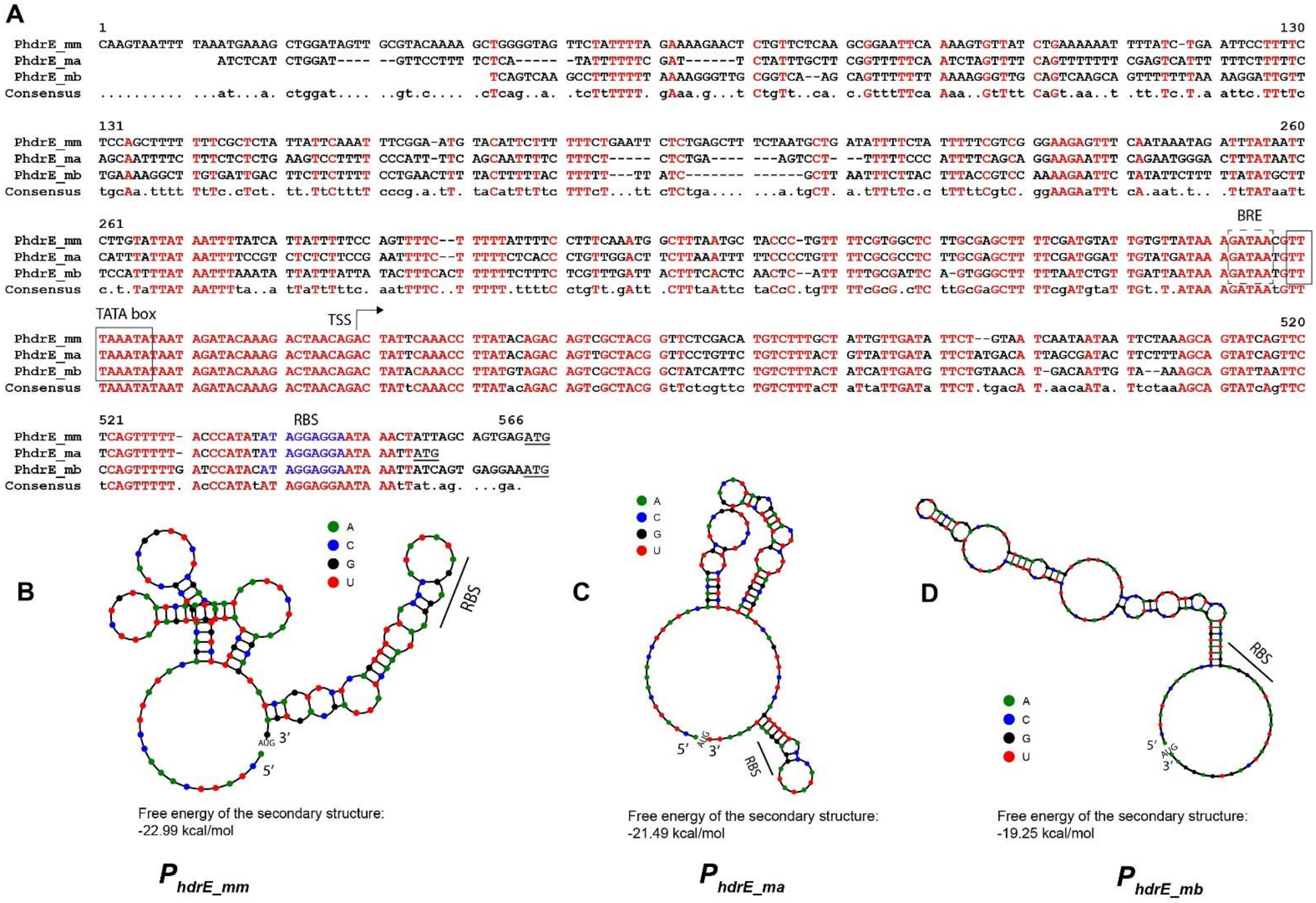

Figure S4. (A) Sequence alignment of *hdrED1* promoter-RBSs from different *Methanosarcina* species. Promoter sequences are derived as follows: P_{hdrE_mm} from *M. mazei*, P_{hdrE_ma} from *M. acetivorans*, and P_{hdrE_mb} from *M. barkeri*. A multiple alignment of DNA sequences was performed with MultAlin (http://multalin.toulouse.inra.fr/multalin/multalin.html) using default settings. Highly conserved regions (> 90% similarity) are indicated (red font). Sequences for the putative promoter elements are indicated: BRE (dashed line bordering), TATA box (solid line bordering), and TSS (black arrow). The putative RBS sequence is shown (purple). The start codon (ATG) is underlined in each DNA sequence. **(B to D) Prediction of 5'UTR secondary structures and their free energies**. RNA secondary structures and their free energies were predicted with NUPACK (https://www.nupack.org/) using default settings. All 5'UTRs are in the 5'-to-3' direction. Bases in the RNA sequence are designated as colored dots (A, green; C, blue; G, black; and U, red). The location of the putative RBS is indicated by a solid line. The location of the start codon (AUG) is shown.

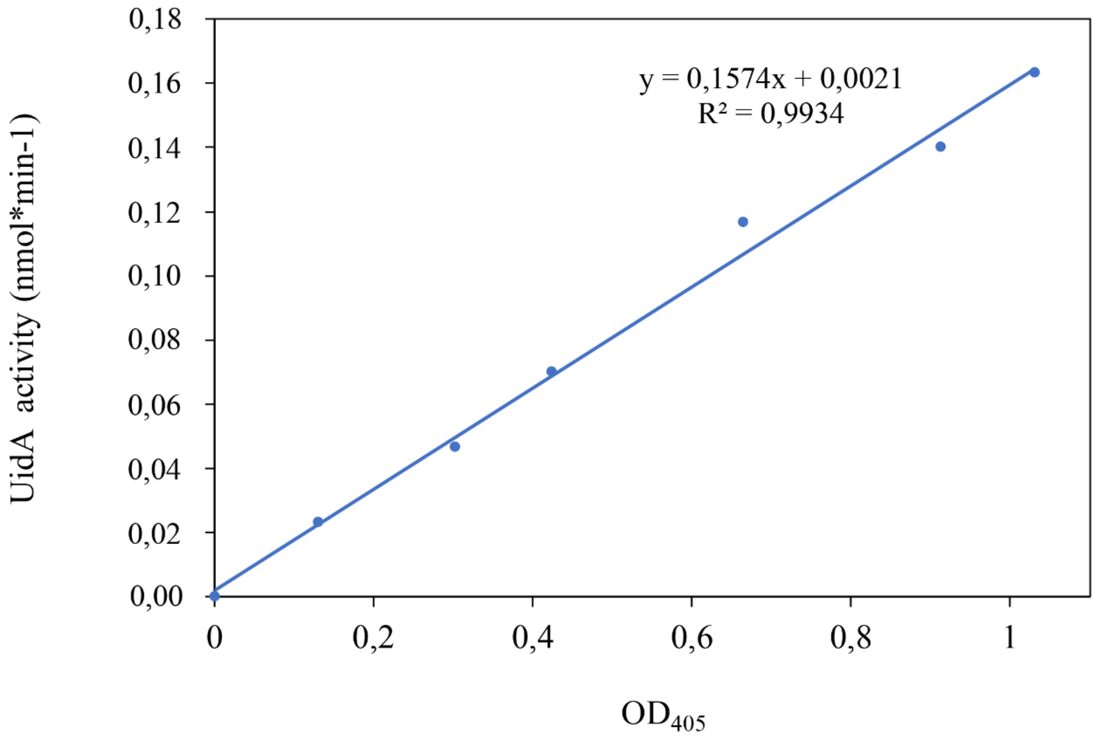

Figure S5. **Standard curve of *E. coli* β-glucuronidase activity.** Activity measurements were performed according to the assay protocol described in Materials and Methods (see main text).

Table S1. Promoter-RBS combinations used in this study[a].

Name	Sequence (5'-to-3' direction)	Length	Source
P_{mcrB_mm}	aaactttcgtataatgaggatattactaaatctttttggtatgattctatgagtgagtattaacag<br>aaccaaaaaaattacattaattcaggcatttctcagaaaaatatataaataagttttttgtcaat<br>tttttgatatttttgggtgtccctaaaaattaaattttcgattgatgctgtttttaatataattatag<br>aaaaatacaattcaccttaataaaatgatttttaaaaaaatacatgaattcatctagcggaga<br>acacaaaagatttaagtaccttctaaacgaatgagatttcattgggaaagtggacacttaaa<br>aacgacgcggtacttgatttattgagtgcaaaagcactcgattaggtgaccagtcccaaag<br>tgattttaataaat**taaggagga**aattaaa	416 bp	This study
P_{mcrB_mb}	aatatcatttcgtcattttcctaagaaatatttttattgggtaatctggtgtatttttgacaggcttt<br>aagtacgttgaaaattcatttttcgatcgatacggtttttatatgtttttataaaaatagattaca<br>aattaataaattatattttaaaaaaatacataaattcatttatcggagaacacaaaagatttaa<br>gtaccttctaaacgaatgagatttcattgggaatagtggacacttaaaaacaaagcggtac<br>ttgatttattgagtgcaaaggcactcgagtaggtgaccagtcccaaaatgattttaataaat**t**<br>**aaggagga**aattaaa	338 bp	This study
P_{mcrB_mv}	aggtaggatattaccaggtatggaattacaaagtataatcaatcgtatgaatatataatattat<br>attactaattcataatactaaaatacgataatttaatacttataggatgatttgtaatacattattg<br>aaaaatttttatatattctcaatacttcagtttagttttaattatttttgaaagtattaaaaaatcttt<br>aaaaaataggggatggtaataaaaaacgccttgtttgtaatatactatcgaaaacgaattatt<br>tatgcgaatattgatataatacttaaattaaatataatatttagatattgggatataataattacat<br>attttaaaaaaatcttctattttcatcttaattttatatattaaaggctaatttttcaatattttatcgc	522 bp	This study

	aaactattgtgttaattacatcaaatatataaacttttctatttaaagctaatgaaaaagtgaat<br>atatacacatagagtaatgttatgatgtatatatcaaaaaa**ataggagtg**attcct		
P$_{mcrB\_mok}$	tatcctctaatgttttattttggttattttagtatccttagtatctcatagttttatgaataaaaataa<br>aataatataatataaaaaataatataattataaatataataaaataaatataaaattttgctaaa<br>actctcaaaaaaccatatattgtaaagaataattaaaaataaataaaattaaattaaaataata<br>aatttaaatatatgaataaatttttataatattcctttaaaaaatatctcctatatatagttttcgat<br>ttgaattcaatggaaaaaggaatatataaataaaaatataggatgtagtgcatatatcaaata<br>**ataggagtg**gttcct	345 bp	This study
P$_{mtrE\_mm}$	aatatattttaaaaaataaataaaaaaaattcattagtaataatcggaatctttatatgatattgaa<br>agaagtaaaatttattctttttatataaatataattttgtttgatttaaccagctcagtacatcata<br>taaaattgattgtataaaatataaacctataataatattttaaaagcgttcctatttcgaaaggtt<br>tatgaatgatcctctctaatccaaaaattaacgtgcgtcataatcatatttctttgtaatgatat<br>ggcagacgcttttaaaaaaatgtacagcgtttttggaatccagtttattacatgtaataacat<br>**aaaggagga**aaatga	344 bp	This study
P$_{mtrE\_mb}$	agtaattatattttaaaaaatgaattcaaaaattcattagttacaatcggaatctttatattatatt<br>gaaaagagtagaatttttttatttatataaatatgaaattttgcttgtttttacctgcgcaatctgt<br>cttataaaattgattatatattatatagtcatataataatattttaaaagcctacatatttcgaaag<br>gtttatgaatgatcctctctaatcaaaaaattaacgcgcgtcataatcatatttctttgtaatga<br>tatggcagacgattttaaaaaaatgtacagcgtttttgaatccaccttattacatgtaataata<br>c**aaaggagga**aaatga	347 bp	This study

P_{hdrE_mm}	caagtaattttaaatgaaagctggatagttgcgtacaaaagctggggtagttctattttagaaaagaactctgttctcaagcggaattcaaaagtgttatctgaaaaaattttatctgaattcctttttctccagcttttttcgctctattattcaaatttcggaatgtacattctttttctgaattctctgagctttctaatgctgatattttctatttttcgtcgggaagagtttcaataaatagatttataattcttgtattataattttatcattatttttccagttttcttttattttcctttcaaatggctttaatgctaccctgttttcgtggctcttgcgagcttttcgatgtattgtgttataaagataacgtttaaatataatagatacaaagactaacagactattcaaaccttatacagacagtcgctacggttctcgacatgtctttgctattgttgatattctgtaatcaataataattctaaagcagtatcagttctcagtttttacccatat**ataggagga**ataaactattagcagtgag	558 bp	This study
P_{hdrE_mb}	ggattgtttgaaaaggcttgtgattgacttcttcttttcctgaacttttactttttactttttttatcgcttaatttcttactttaccgtccaaaagaattctatattctttttatatgctttccattttataatttaaatattatttattatactttcactttttctttctcgtttgattactttcactcaactcatttttgcgattcagtgggctttttaatctgttgattaataaagataatgtttaaatataatagatacaaagactaacagactatacaaaccttatgtagacagtcgctacggctatcattctgtctttactatcattgatgttctgtaacatgacaattgtaaaagcagtattaattcccagtttttgatccatac**ataggagga**ataaattatcagtgaggaa	422 bp	This study
P_{acs_mb}	ctaatctctttttgtacctataattacaataaacttgtaaagtcaactgattttgaacattcccactttacccatctcgaaatgcgtgttacaaattagaaaatattatatagatttggtacgttaagacatttaattagtgtttattatccatcggtagcgacctctgcttaggattaaggtctcaatcgttggcaaaaacggttttttgagacaaagggcgtaaaaatggctttgaagtgtgaataagccctaaaaacgacttttatatggaaatatttcctctaaaaacgactttagctggaaaaacgctggtaaaatatttgttatttgaaaaaaagtggtgaatagtgttttaccagatgcgtatttacttacacgg	501 bp	This study

	ttgtaactttaagcagacggcaaggagtgtttggtaattacaaatatgccgaagaattaaaa<br>attgcaatcgtcttgatgaagtcgactttatacaaat**ttaggaggt**aaagctcaa		
P_{ech_mb}	tcagtatgtttaaatatagctttgttcatgctaatttatactacttaagtagtactgggattatttg<br>gtagtatttattaacactcagtaatatttataacgaggatgtaaacaagaaaaaaacaatattt<br>gggggctattttgtccattattctcgttagtaatataaaagtaaaaagaaaatcactatctttaa<br>atatgcctttgttataataagaattggcaacatgtcgaatttatatcgacattattcgcatattat<br>tatttcttgtaattttgatataagaatactataatatttgtggaatattagtccatattcttatatact<br>caggaattgcgcagttgaactggaaaatcaatatatcaagtcattcgagtatatagaaaata<br>ggttacaaaaacgtccaagatattggattctgtttttcagctgcataagttccagcatcataa<br>gtcagagaataacagatcggaggattaatag**atcggagga**tacgctgt	504 bp	This study
P_{vhxG_mb}	tccaaaaatattaaagcctgttgaactttccttaccgcagagccacggtcccctctttggag<br>atatctttcgttgtcataacaagcttccagaacctcgtcgccttcaaatctcgacgaccttaa<br>atcgtgaccatccccaatagtcatattctatcccccacgtttgccgcataacttttcccttaaa<br>tatgcaacaataacttttcccttaaatatgcaacattatcactaaatactactagccttaaaattt<br>caataaaattccaataaataaactagtgatattaaaatatataagtttttagaaaaattatattta<br>ttaaatgatttaaatttaatttaaacaatctcctactgttgcaaagtaaaggagttacttgtgaa<br>gtggttattgaaggtttgatagtctttgatatatatgtgatattctttaatatattggattctatttttt<br>tatataccatttttatatatctttatatgaaattattagtaac	500 bp	This study
P_{atpH_mm}	cgcagtgagagatatgttttcttactaattcttaatttgaaatttattttacaaagtcttacgtcaa<br>ttcagtaacttcttatttattttaaaaagatccttaaaagcatctcctgaatacaggtttggaga<br>atagatttattccgtactctgatcttttttttggtggtgaaacctgatttttatcatttaattatatt<br>ctactgtttctatttaattatttttactgttatatttaatcttttggattttaaccatatattttgttattt	526 bp	This study

	atattttttaatttcttaataatttcttgcaaatttaatatatcatgaataatcattacagttattttaa<br>tcctcttcgataaaaccgaagctgttatatagttctttcacgaaggatttgttaactacctccat<br>tggagtagggatttttaattattaatttatatattaaatttttg**agagacgga**gattcacatggct<br>aaaaatgaaatcttatccgaaataaaaaaagcggaagagagcgctaaatta		
P_{atpH_mb}	tctcttacagttttggtcaaataaagcaacgtatatttttgcttaaaaaagccttcaaaagtttt<br>tatttaggaaaaaagatgctaaaattccttatataaattgattcatttataatttttatctgattta<br>atatagttttgaatatattctccagattttacttaacttattcaggttactcttcacattttattttcg<br>gcactttctttgatatttttcggtacttaattatatttcactgtcagtgttcaattattttttactgttat<br>atttaatctttttcattttaaccggatattttgctatttatatttttaattttcttaaaaatttcttataca<br>ttgaatatataatgatttttcattatagttctcccactcttctttgataaaaccgaagctgttatat<br>agttctttcgcgaaggatttgttaactacctccattggagtagggatttttaattattaatttata<br>tattaaatttttg**agagacgga**gattcac	508 bp	This study
P_{atpH_ma}	tcactttaaatatcttaactgtgaaaaaggggaatatgtgcgttttaaggctaaatacacaca<br>ttggatatattttacaaagtcttaaattcaggatgtaaattcacatttatttttgactgaactcaa<br>aatcccctattaaaacattaattcaaaaaagttctttcatggctttgcccttctttatgcgggca<br>ggactcggcatttttttatcaattaattatattcaactgtttctatttaattattttttactgttctattta<br>atcttttggattttaaccatatattttgatatttatatttttaaaattcttaaaaatttcctgcacattc<br>agtacataatgattgatcgtgataattattaatgtcctcttcgataaaaccgaagctgttatat<br>agttctttcgcaaaggatttgttaactacctccattggagtagggatttttaattattaatttatat<br>attaaatttttg**agagacgga**gattcac	493 bp	This study

minimal P_{mcrB}	caaaagatttaagtaccttctaaacgaatgagatttcattgggaatagtggacactcgagta<br>ggtgaccagtcccaaaatgattttaataaat**taaggagga**aattca	127 bp	This study
P_{hdrE_mb}-1[b]	ggattgtttgaaaaggcttgtgattgacttcttcttttcctgaacttttactttttactttttttatcg<br>cttaatttcttactttaccgtccaaaagaattctatattctttttatatgctttccattttataatttaa<br>atattatttattatactttcactttttttctttctcgtttgattactttcactcaactcatttttgcgattc<br>agtgggctttttaatctgttgattaataaagataatgtttaaatataatagatacaaagactaa<br>cagactatacaaaccttatgtagacagtcgctacggctatcattctgtctttactatcattgat<br>gttctgtaacatgacaattgtaaaagcagtattaattcccagtttttgatccattgattttaataa<br>at**taaggagga**aattaaa	422 bp	This study
P_{hdrE_mb}-2[b]	aatatcatttcgtcattttcctaagaaatatttttattgggtaatctggtgtatttttgacaggcttt<br>aagtacgttgaaaattcatttttcgatcgatacggtttttttatatgtttttataaaaatagattaca<br>aattaataaattatattttaaaaaaatacataaattcatttatcggagaacacaaaagatttaa<br>gtaccttctaaacgaatgagatttcattgggaatagtggacacttaaaaacaaagcggtac<br>ttgatttattgagtgcaaaggcactcgagtaggtgaccagtcccaaaaac**ataggagga**a<br>taaattatcagtgaggaa	338 bp	This study
P_{mtrE_mb}-1[b]	agtaattatattttaaaaaaatgaattcaaaaattcattagttacaatcggaatctttatattatatt<br>gaaaagagtagaatttttttatttatataaatatgaaattttgcttgtttttacctgcgcaatctgt<br>cttataaaattgattatatattatatagtcatataataatattttaaaagcctacatatttcgaaag<br>gtttatgaatgatcctctctaatcaaaaaattaacgcgcgtcataatcatatttctttgtaatga<br>tatggcagacgattttaaaaaaatgtacagcgtttttttgaatccaccttattgattttaataaat**t**<br>**aaggagga**aattaaa	347 bp	This study
P_{mtrE_mb}-2[b]	aatatcatttcgtcattttcctaagaaatatttttattgggtaatctggtgtatttttgacaggcttt<br>aagtacgttgaaaattcatttttcgatcgatacggtttttttatatgtttttataaaaatagattaca<br>aattaataaattatattttaaaaaaatacataaattcatttatcggagaacacaaaagatttaa<br>gtaccttctaaacgaatgagatttcattgggaatagtggacacttaaaaacaaagcggtac<br>ttgatttattgagtgcaaaggcactcgagtaggtgaccagtcccaaaatacatgtaataata<br>c**aaaggagga**aaatga	338 bp	This study
P_{vhxG_mb}-1[b]	tccaaaaatattaaagcctgttgaactttccttaccgcagagccacggtcccctctttggag<br>atatctttcgttgtcataacaagcttccagaacctcgtcgccttcaaatctcgacgaccttaa<br>atcgtgaccatccccaatagtcatattctatcccccacgtttgccgcataacttttcccttaaa<br>tatgcaacaataacttttcccttaaatatgcaacattatcactaaatactactagccttaaaattt<br>caataaaattccaataaataaactagtgatattaaaatatataagtttttagaaaaattatattta<br>ttaaatgatttaaatttaatttaaacaatctcctactgttgcaaagtaaaggagttacttgtgaa<br>gtggttattgaaggtttgatagtctttgatatatatgtgatattctttaatatattggattctatttttt<br>tatataccatttttttgattttaataaat**taaggagga**aattaaa	500 bp	This study
P_{vhxG_mb}-2[b]	aatatcatttcgtcattttcctaagaaatatttttattgggtaatctggtgtatttttgacaggcttt<br>aagtacgttgaaaattcatttttcgatcgatacggtttttttatatgtttttataaaaatagattaca<br>aattaataaattatattttaaaaaaatacataaattcatttatcggagaacacaaaagatttaa<br>gtaccttctaaacgaatgagatttcattgggaatagtggacacttaaaaacaaagcggtac	338 bp	This study

	ttgatttattgagtgcaaaggcactcgagtaggtgaccagtcccaaaatatatatctttatatgaaattattagtaac		
P_{acs_mb}-1[b]	ctaatctctttttttgtacctataattacaataaacttgtaaagtcaactgattttgaacattcccactttacccatctcgaaatgcgtgttacaaattagaaaatattatatagatttggtacgttaagacatttaattagtgtttattatccatcggtagcgacctctgcttaggattaaggtctcaatcgttggcaaaaacggtttttttgagacaaagggcgtaaaaatggctttgaagtgtgaataagccctaaaaacgacttttatatggaaatatttcctctaaaaacgactttagctggaaaaacgctggtaaaatatttgttatttgaaaaaaagtggtgaatagtgttttaccagatgcgtatttacttacacggttgtaactttaagcagacggcaaggagtgtttggtaattacaaatatgccgaagaattaaaaattgcaatcgtcttgatgaagtcgatgattttaataaat**taaggagga**aattaaa	501 bp	This study
P_{acs_mb}-2[b]	aatatcatttcgtcattttcctaagaaatatttttattgggtaatctggtgtatttttgacaggctttaagtacgttgaaaattcatttttcgatcgatacggtttttttatatgtttttataaaaatagattacaaattaataaattatattttaaaaaaatacataaattcatttatcggagaacacaaaagatttaagtaccttctaaacgaatgagatttcattgggaatagtggacacttaaaaacaaagcggtacttgatttattgagtgcaaaggcactcgagtaggtgaccagtcccaaaactttatacaaat**ttaggaggt**aaagctcaa	338 bp	This study
P_{ech_mb}-1[b]	tcagtatgtttaaatatagctttgttcatgctaatttatactacttaagtagtactgggattatttggtagtatttattaacactcagtaatatttataacgaggatgtaaacaagaaaaaaacaatatttgggggctattttgtccattattctcgttagtaatataaaagtaaaaagaaaatcactatctttaaatatgcctttgttataataagaattggcaacatgtcgaatttatatcgacattattcgcatattattatttcttgtaattttgatataagaatactataatatttgtggaatattagtccatattcttatatactcaggaattgcgcagttgaactggaaaatcaatatatcaagtcattcgagtatatagaaaataggttacaaaaacgtccaagatattggattctgtttttcagctgcataagttccagcatcataagtcagagaataacagatctgattttaataaat**taaggagga**aattaaa	504 bp	This study
P_{ech_mb}-2[b]	aatatcatttcgtcattttcctaagaaatatttttattgggtaatctggtgtatttttgacaggctttaagtacgttgaaaattcatttttcgatcgatacggtttttttatatgtttttataaaaatagattacaaattaataaattatattttaaaaaaatacataaattcatttatcggagaacacaaaagatttaagtaccttctaaacgaatgagatttcattgggaatagtggacacttaaaaacaaagcggtacttgatttattgagtgcaaaggcactcgagtaggtgaccagtcccaaaaggaggattaatag**atcggagga**tacgctgt	338 bp	This study
P_{mcrB_mok}-1[b]	tatcctctaatgttttattttggttattttagtatccttagtatctcatagttttatgaataaaaataaaataatataatataaaaaaataatataattataaatataataaaataaatataaaattttgctaaaactctcaaaaaaccatatattgtaaagaataattaaaaataaataaaattaaattaaaataataaatttaaatatatgaataaattttttataatattcctttaaaaaatatctcctatatatagttttcgatttgaattcaatggaaaaaggaatatataaataaaaatataggatgtagtgattttaataaat**taaggagga**aattaaa	345 bp	This study
P_{mcrB_mok}-2[b]	aatatcatttcgtcattttcctaagaaatatttttattgggtaatctggtgtatttttgacaggctttaagtacgttgaaaattcatttttcgatcgatacggtttttttatatgtttttataaaaatagattacaaattaataaattatattttaaaaaaatacataaattcatttatcggagaacacaaaagatttaagtaccttctaaacgaatgagatttcattgggaatagtggacacttaaaaacaaagcggtacttgatttattgagtgcaaaggcactcgagtaggtgaccagtcccaaaatgcatatatcaaata**ataggagtg**gttcct	338 bp	This study

P_{mcrB_mv}-1[b]	aggtaggatattaccaggtatggaattacaaagtataatcaatcgtatgaatatataatattat<br>attactaattcataatactaaaatacgataatttaatacttataggatgatttgtaatacattattg<br>aaaaatttttatatattctcaatacttcagtttagttttaattatttttgaaagtattaaaaaatcttt<br>aaaaaataggggatggtaataaaaaacgccttgtttgtaatatactatcgaaaacgaattatt<br>tatgcgaatattgatataatacttaaattaaatataatatttagatattgggatataataattacat<br>attttaaaaaaatcttctattttcatcttaattttatatattaaaggctaatttttcaatattttatcgc<br>aaactattgtgttaattacatcaaatatataaactttttctatttaaagctaatgaaaaagtgaat<br>atatacacatagagtaatgttatgattgattttaataaatt**taaggagga**aattaaa	522 bp	This study
P_{mcrB_mv}-2[b]	aatatcatttcgtcattttcctaagaaatatttttattgggtaatctggtgtatttttgacaggcttt<br>aagtacgttgaaaattcatttttcgatcgatacggtttttatatgtttttataaaaatagattaca<br>aattaataaattatattttaaaaaaatacataaattcatttatcggagaacacaaaagatttaa<br>gtaccttctaaacgaatgagatttcattgggaatagtggacttaaaaacaaagcggtac<br>ttgatttattgagtgcaaaggcactcgagtaggtgaccagtcccaaaagtatatatcaaaaa<br>a**ataggagtg**attcct	338 bp	This study
P_{UTR1} [c]	aaactttcgtataatgaggatattactaaatctttttggtatgattctatgagtgagtattaacag<br>aaccaaaaaaaattacattaattcaggcatttctcagaaaaatatataaataagtttttgtcaat<br>tttttgatatttttgggtgtccctaaaaattaaattttcgattgatgctgtttttaatataattatag<br>aaaaatacaattcaccttaataaaatgatttttaaaaaaatacatgaattcatctagcggaga<br>acacaaaagatttaagtaccttctaaacgaatgagatttcattgggaaagtggacacttaaa<br>aacgacgcggtacttgatttattgagtgcaaaagcactcgattaggtgaccagtcccaaag<br>tgattttaataaatt**Aaggagga**aattaaa	417 bp	This study
P_{UTR2} [c]	aaactttcgtataatgaggatattactaaatctttttggtatgattctatgagtgagtattaacag<br>aaccaaaaaaaattacattaattcaggcatttctcagaaaaatatataaataagtttttgtcaat<br>tttttgatatttttgggtgtccctaaaaattaaattttcgattgatgctgtttttaatataattatag<br>aaaaatacaattcaccttaataaaatgatttttaaaaaaatacatgaattcatctagcggaga<br>acacaaaagatttaagtaccttctaaacgaatgagatttcattgggaaagtggacacttaaa<br>aacgacgcggtacttgatttattgagtgcaaaagcactcgattaggtgaccagtcccaaag<br>tgattt~~**t**~~aataa**a**tt**Aaggagga**aattaaa	415 bp	This study
P_{UTR3} [c]	aaactttcgtataatgaggatattactaaatctttttggtatgattctatgagtgagtattaacag<br>aaccaaaaaaaattacattaattcaggcatttctcagaaaaatatataaataagtttttgtcaat<br>tttttgatatttttgggtgtccctaaaaattaaattttcgattgatgctgtttttaatataattatag<br>aaaaatacaattcaccttaataaaatgatttttaaaaaaatacatgaattcatctagcggaga<br>acacaaaagatttaagtaccttctaaacgaatgagatttcattgggaaagtggacacttaaa<br>aacgacgcggtacttgatttattgagtgcaaaagcactcgattaggtgaccagtcccaaag<br>t~~**gattttaata**~~aatt**Taaggagga**~~**a**~~attaaa**T**	408 bp	This study
P_{UTR4} [c]	aaactttcgtataatgaggatattactaaatctttttggtatgattctatgagtgagtattaacag<br>aaccaaaaaaaattacattaattcaggcatttctcagaaaaatatataaataagtttttgtcaat<br>tttttgatatttttgggtgtccctaaaaattaaattttcgattgatgctgtttttaatataattatag<br>aaaaatacaattcaccttaataaaatgatttttaaaaaaatacatgaattcatctagcggaga<br>acacaaaagatttaagtaccttctaaacgaatgagatttcattgggaaagtggacacttaaa<br>aacgacgcggtacttgatttattgagtgcaaaagcactcgattaggtgaccagtcccaaag<br>tgattt~~**t**~~aataa~~**a**~~**T**t**taaggagga**~~**a**~~attaaa**T**	415 bp	This study

P_{UTR5} [c]	aaactttcgtataatgaggatattactaaatctttttggtatgattctatgagtgagtattaacag<br>aaccaaaaaaaattacattaattcaggcatttctcagaaaaatatataaataagtttttgtcaat<br>tttttgatatttttgggtgtccctaaaaattaaattttcgattgatgctgtttttaatataattatag<br>aaaaatacaattcaccttaataaaatgatttttaaaaaaatacatgaattcatctagcggaga<br>acacaaaagatttaagtaccttctaaacgaatgagatttcattgggaaagtggacacttaaa<br>aacgacgcggtacttgatttattgagtgcaaaagcactcgattaggtgaccagtcccaaag<br>~~**tgat**~~tttaataa~~**a**~~**T**t**taaggagga**~~**a**~~attaaa**T**	412 bp	This study
P_{UTR6} [c]	aaactttcgtataatgaggatattactaaatctttttggtatgattctatgagtgagtattaacag<br>aaccaaaaaaaattacattaattcaggcatttctcagaaaaatatataaataagtttttgtcaat<br>tttttgatatttttgggtgtccctaaaaattaaattttcgattgatgctgtttttaatataattatag<br>aaaaatacaattcaccttaataaaatgatttttaaaaaaatacatgaattcatctagcggaga<br>acacaaaagatttaagtaccttctaaacgaatgagatttcattgggaaagtggacacttaaa<br>aacgacgcggtacttgatttattgagtgcaaaagcactcgattaggtgaccagtcccaaag<br>tgattttaataaat**TTAGGAGG**taattaaa	416 bp	This study

[a] Putative elements for all promoter-RBS combinations are indicated: BRE (dashed underlining), TATA box (solid line bordering), TSS (highlighted in magenta), and **RBS** (boldface font highlighted in gray). Putative 16s rRNA standby site is indicated (double underlining).

[b] For promoter/RBS-hybrid combinations: the exchanged RBS region (RBS_{mcr}) of the wild-type promoter-RBS combination via V1 strategy (see **Figure 2A**) is marked with wave underline, and the exchanged promoter region via the V2 strategy is underlined.

[c] For the 5'UTR-engineered combinations: mutated (enlarged uppercase boldface font) and deleted (strikethrough boldface font) bases are indicated.

Table S2. Primers used in this study.

Primer	Sequence (5'-to-3' direction)	Source	Description
L730-F	ggatccaagcttgggccctcgcga	This study	Linearize730-F
L730-R	gcgagggcccaagcttggatccgggagaattatatgagct tataacggtagaaatattgtttgat	This study	Linearize730-R
P0-F	gcatctgtgcggtatttcacacctcatatgttacgtcctgtag aaaccccaacc	This study	uid cassette-F
P0-R	gcgagggcccaagcttggatccgggagaattatatgagct tataacggtagaaatattgtttgat	This study	uid cassette-R
P1-F	gcatctgtgcggtatttcacacctcataaactttcgtataatga ggatattactaaat	This study	P_{mcrB_mm}-F
P1-R	ggtttctacaggacgtaacattttaatttcctccttaatttattaa aatcactttg	This study	P_{mcrB_mm}-R
P2-F	gcatctgtgcggtatttcacacctcataatatcatttcgtcattt tcctaagaaatatttttattgg	This study	P_{mcrB_mb}-F
P2-R	ggttggggtttctacaggacgtaacattttaatttcctccttaa tttattaaaatcattttgggac	This study	P_{mcrB_mb}-R
P3-F	gtgcggtatttcacacctcataggtaggatattaccaggtat ggaattaca	This study	P_{mcrB_mv}-F
P3-R	ggtttctacaggacgtaacataggaatcactcctatttttttga tatatacatcat	This study	P_{mcrB_mv}-R
P4-F	ctgtgcggtatttcacacctcattatcctctaatgttttattttgg ttattttagtatc	This study	P_{mcrB_mok}-F
P4-R	ggtttctacaggacgtaacataggaaccactcctattatttga tatatg	This study	P_{mcrB_mok}-R
P5-F	ctgtgcggtatttcacacctcataatatattttaaaaaataaat aaaaaaattcattagtaataatcgg	This study	P_{mtrE_mm}-F
P5-R	ggtttctacaggacgtaacattcattttcctcctttatgttattac atgtaataaac	This study	P_{mtrE_mm}-R
P6-F	ctgtgcggtatttcacacctcatagtaattatattttaaaaaat gaattcaaaaattcattagttac	This study	P_{mtrE_mb}-F
P6-R	ttggggtttctacaggacgtaacattcattttcctcctttgtatt attacatgtaat	This study	P_{mtrE_mb}-R
P7-F	ctgtgcggtatttcacacctcatcaagtaattttaaatgaaag ctggatagttg	This study	P_{hdrE_mm}-F
P7-R	ggtttctacaggacgtaacatctcactgctaatagtttattcct cc	This study	P_{hdrE_mm}-R
P8-F	tctgtgcggtatttcacacctcattcagtcaagcctttttttaaa agggttg	This study	P_{hdrE_mb}-F
P8-R	ttggggtttctacaggacgtaacatttcctcactgataatttatt cctcctatgt	This study	P_{hdrE_mb}-R

P9-F	tgtgcggtatttcacacctcatctaatctctttttgtacctataattacaataaacttgt	This study	P_{acs_mb}-F
P9-R	ttggggtttctacaggacgtaacatttgagctttacctcctaaatttgtataaagtcg	This study	P_{acs_mb}-R
P10-F	tctgtgcggtatttcacacctcattcagtatgtttaaatatagcttgttcatgct	This study	P_{ech_mb}-F
P10-R	ttggggtttctacaggacgtaacatacagcgtatcctccgatctattaa	This study	P_{ech_mb}-R
P11-F	tgtgcggtatttcacacctcattccaaaaatattaaagcctgttgaactttcctt	This study	P_{vhxG_mb}-F
P11-R	ttggggtttctacaggacgtaacatgttactaataatttcatataaagatatataaaaaatggta	This study	P_{vhxG_mb}-R
P12-F	tctgtgcggtatttcacacctcatcttcatttatcggagaacacaaaagatttaag	This study	*minimal* P_{mcrB}-F
P12-R	ttggggtttctacaggacgtaacattgaatttcctccttaatttattaaaatcattttgggac	This study	*minimal* P_{mcrB}-R
P13-F	tctgtgcggtatttcacacctcatggattgtttgaaaaggcttgtga	This study	P_{hdrE_mb}-1-F
P13-R	cgtaacattttaatttcctccttaatttattaaaatcaatggatcaaaaactgggaattaatactg	This study	P_{hdrE_mb}-1-R
P14-R	ttggggtttctacaggacgtaacatttcctcactgataatttattcctcctatgtttttgggactggtcacctactcg	This study	P_{hdrE_mb}-2-R
P15-F	ctgtgcggtatttcacacctcatagtaattatattttaaaaaatgaattcaaaaattcattagttac	This study	P_{mtrE_mb}-1-F
P15-R	cgtaacattttaatttcctccttaatttattaaaatcaataaggtggattcaaaaaacgctg	This study	P_{mtrE_mb}-1-R
P16-R	ttggggtttctacaggacgtaacattcattttcctcctttgtattattacatgtattttgggactggtcacctactcg	This study	P_{mtrE_mb}-2-R
P17-F	tgtgcggtatttcacacctcattccaaaaatattaaagcctgttgaactttcctt	This study	P_{vhxG_mb}-1-F
P17-R	cgtaacattttaatttcctccttaatttattaaaatcaaaaaatggtatataaaaaaatagaatccaatatattaaag	This study	P_{vhxG_mb}-1-R
P18-R	ttggggtttctacaggacgtaacatgttactaataatttcatataaagatatatattttgggactggtcacctactcg	This study	P_{vhxG_mb}-2-R
P19-F	tgtgcggtatttcacacctcatctaatctctttttgtacctataattacaataaacttgt	This study	P_{acs_mb}-1-F

P19-R	cgtaacattttaatttcctccttaatttattaaaatcatcgacttcatcaagacgattgcaa	This study	P_{acs_mb}-1-R
P20-R	ttggggtttctacaggacgtaacatttgagctttacctcctaaatttgtataaagttttgggactggtcacctactcg	This study	P_{acs_mb}-2-R
P21-F	tctgtgcggtatttcacacctcattcagtatgtttaaatatagctttgttcatgct	This study	P_{ech_mb}-1-F
P21-R	cgtaacattttaatttcctccttaatttattaaaatcagatctgttattctctgacttatgatgc	This study	P_{ech_mb}-1-R
P22-R	ttggggtttctacaggacgtaacatacagcgtatcctccgatctattaatcctccttttgggactggtcacctactcg	This study	P_{ech_mb}-2-R
P23-F	tgtgcggtatttcacacctcattatcctctaatgttttattttggttattttagtatcc	This study	P_{mcrB_mok}-1-F
P23-R	cgtaacattttaatttcctccttaatttattaaaatcactacatcctatatttttatttatatattcctttttccat	This study	P_{mcrB_mok}-1-R
P24-R	ttggggtttctacaggacgtaacataggaaccactcctattatttgatatatgcattttgggactggtcacctactcg	This study	P_{mcrB_mok}-2-R
P25-F	gtgcggtatttcacacctcataggtaggatattaccaggtatggaattaca	This study	P_{mcrB_mv}-1-F
P25-R	cgtaacattttaatttcctccttaatttattaaaatcaatcataacattactctatgtgtatatattcactttttc	This study	P_{mcrB_mv}-1-R
P26-R	ttggggtttctacaggacgtaacataggaatcactcctatttttttgatatatacttttgggactggtcacctactcg	This study	P_{mcrB_mv}-2-R
P27-F	acggtctcactcataaactttcgtataatgaggatattactaaatctttttgg	This study	P_{UTR1} -F (Golden Gate Assembly)
P27-R	atggtctcaacattttaatttcctcctttaatttattaaaatcactttgggactggtc	This study	P_{UTR1} -R (Golden Gate Assembly)
P28-R	atggtctcaacattttaatttcctcctttaatttattaaaatcactttgggactggtc	This study	P_{UTR2} -R (Golden Gate Assembly)
P29-R	atggtctcaacattttaatttcctcctttaatttattaaaatcactttgggactggtc	This study	P_{UTR3} -R (Golden Gate Assembly)
P30-R	atggtctcaacatatttaattcctccttaaattattaaatcactttgggactggtcacctaatcg	This study	P_{UTR4} -R (Golden Gate Assembly)
P31-R	atggtctcaacatatttaattcctccttaaattattaaactttgggactggtcacctaatcg	This study	P_{UTR5} -R (Golden Gate Assembly)
P32-R	atggtctcaacattttaattacctcctaaatttattaaaatcactttgggactggtc	This study	P_{UTR6} -R (Golden Gate Assembly)

P33-F	tgtgcggtatttcacacctcatcgcagtgagagatatgttttcttacta	This study	P_{atpH_mm} -F
P33-R	ttggggtttctacaggacgtaacattaatttagcgctctcttccgcttttttta	This study	P_{atpH_mm} -R
P34-F	gtgcggtatttcacacctcattctcttacagttttggtcaaataaagca	This study	P_{atpH_mb} -F
P34-R	ttggggtttctacaggacgtaacatgtgaatctccgtctctcaaaatttaatat	This study	P_{atpH_mb} -R
P35-F	gtgcggtatttcacacctcattcactttaaatatcttaactgtgaaaaaggggaa	This study	P_{atpH_ma} -F
P35-R	ttggggtttctacaggacgtaacatgtgaatctccgtctctcaaaatttaat	This study	P_{atpH_ma} -R
**Colony PCR**	**Sequence**	**Source**	**Description**
P36	cctggcgttacccaacttaatc	This study	verify730-F
P37	gattcattctagtcccgtcagg	This study	verify730-R

Table S3. Strains and plasmids used in this study.

Strain	Genotype	Plasmid	Description	Source
WWM73	Δ*hpt*::P*mcrB-tetR*-φC31-*int*-attP	none	none	(2)
		pNB730	*pUC19 ori, bla*$^{+}$, *lacZ*$^{+}$, φC31 *attB*, PmcrB (*M. voltae*) *pac (opt), strep-his MCS his-strep* expression cassette	(3)
PZ0	WWM73::pPZ00	pPZ00[a]	pNB730-derived plasmid with *uidA* cassette (*uidA* fused with Tmcr)	This study
PZ1	WWM73::pPZ01	pPZ01[a]	pNB730-derived plasmid with P_{mcrB_mm} - *uidA* cassette	This study
PZ2	WWM73::pPZ02	pPZ02[a]	pNB730-derived plasmid with P_{mcrB_mb} - *uidA* cassette	This study
PZ3	WWM73::pPZ03	pPZ03[a]	pNB730-derived plasmid with P_{mcrB_mv} - *uidA* cassette	This study
PZ4	WWM73::pPZ04	pPZ04[a]	pNB730-derived plasmid with P_{mcrB_mok} - *uidA* cassette	This study
PZ5	WWM73::pPZ05	pPZ05[a]	pNB730-derived plasmid with P_{mtrE_mm} - *uidA* cassette	This study
PZ6	WWM73::pPZ06	pPZ06[a]	pNB730-derived plasmid with P_{mtrE_mb} - *uidA* cassette	This study
PZ7	WWM73::pPZ07	pPZ07[a]	pNB730-derived plasmid with P_{hdrE_mm} - *uidA* cassette	This study
PZ8	WWM73::pPZ08	pPZ08[a]	pNB730-derived plasmid with P_{hdrE_mb} - *uidA* cassette	This study
PZ9	WWM73::pPZ09	pPZ09[a]	pNB730-derived plasmid with P_{acs_mb} - *uidA* cassette	This study
PZ10	WWM73::pPZ10	pPZ10[a]	pNB730-derived plasmid with P_{ech_mb} - *uidA* cassette	This study
PZ11	WWM73::pPZ11	pPZ11[a]	pNB730-derived plasmid with P_{vhxG_mb} - *uidA* cassette	This study
PZ12	WWM73::pPZ12	pPZ12[a]	pNB730-derived plasmid with *minimal* P_{mcrB} - *uidA* cassette	This study
PZ13	WWM73::pPZ13	pPZ13[a]	pNB730-derived plasmid with P_{hdrE_mb}-1- *uidA* cassette	This study
PZ14	WWM73::pPZ14	pPZ14[a]	pNB730-derived plasmid with P_{hdrE_mb}-2 - *uidA* cassette	This study
PZ15	WWM73::pPZ15	pPZ15[a]	pNB730-derived plasmid with P_{mtrE_mb}-1 - *uidA* cassette	This study
PZ16	WWM73::pPZ16	pPZ16[a]	pNB730-derived plasmid with P_{mtrE_mb}-2 - *uidA* cassette	This study
PZ17	WWM73::pPZ17	pPZ17[a]	pNB730-derived plasmid with P_{vhxG_mb}-1 - *uidA* cassette	This study

PZ18	WWM73::pPZ18	pPZ18[a]	pNB730-derived plasmid with P_{vhxG_mb}-2 - *uidA* cassette	This study
PZ19	WWM73::pPZ19	pPZ19[a]	pNB730-derived plasmid with P_{acs_mb}-1 - *uidA* cassette	This study
PZ20	WWM73::pPZ20	pPZ20[a]	pNB730-derived plasmid with P_{acs_mb}-2 - *uidA* cassette	This study
PZ21	WWM73::pPZ21	pPZ21[a]	pNB730-derived plasmid with P_{ech_mb}-1 - *uidA* cassette	This study
PZ22	WWM73::pPZ22	pPZ22[a]	pNB730-derived plasmid with P_{ech_mb}-2 - *uidA* cassette	This study
PZ23	WWM73::pPZ23	pPZ23[a]	pNB730-derived plasmid with P_{mcrB_mok}-1 - *uidA* cassette	This study
PZ24	WWM73::pPZ24	pPZ24[a]	pNB730-derived plasmid with P_{mcrB_mok}-2 - *uidA* cassette	This study
PZ25	WWM73::pPZ25	pPZ25[a]	pNB730-derived plasmid with P_{mcrB_mv}-1 - *uidA* cassette	This study
PZ26	WWM73::pPZ26	pPZ26[a]	pNB730-derived plasmid with P_{mcrB_mv}-2 - *uidA* cassette	This study
PZ27	WWM73::pPZ27	pPZ27[a]	pNB730-derived plasmid with P_{UTR1} - *uidA* cassette	This study
PZ28	WWM73::pPZ28	pPZ28[a]	pNB730-derived plasmid with P_{UTR2} - *uidA* cassette	This study
PZ29	WWM73::pPZ29	pPZ29[a]	pNB730-derived plasmid with P_{UTR3}- *uidA* cassette	This study
PZ30	WWM73::pPZ30	pPZ30[a]	pNB730-derived plasmid with P_{UTR4} - *uidA* cassette	This study
PZ31	WWM73::pPZ31	pPZ31[a]	pNB730-derived plasmid with P_{UTR5} - *uidA* cassette	This study
PZ32	WWM73::pPZ32	pPZ32[a]	pNB730-derived plasmid with P_{UTR6} - *uidA* cassette	This study
PZ33	WWM73::pPZ33	pPZ33[a]	pNB730-derived plasmid with P_{atpH_mm} - *uidA* cassette	This study
PZ34	WWM73::pPZ34	pPZ34[a]	pNB730-derived plasmid with P_{atpH_mb} - *uidA* cassette	This study
PZ35	WWM73::pPZ35	pPZ35[a]	pNB730-derived plasmid with P_{atpH_ma} - *uidA* cassette	This study

[a] The plasmid sequence is available by clicking the link.

References

1. Rohlin L, Gunsalus RP. 2010. Carbon-dependent control of electron transfer and central carbon pathway genes for methane biosynthesis in the Archaean, *Methanosarcina acetivorans* strain C2A. *BMC Microbiol* 10:62.

2. Guss AM, Rother M, Zhang JK, Kulkkarni G, Metcalf WW. 2008. New methods for tightly regulated gene expression and highly efficient chromosomal integration of cloned genes for *Methanosarcina* species. *Archaea* 2:193–203.

3. Shea MT, Walter ME, Duszenko N, Ducluzeau A-L, Aldridge J, King SK, Buan NR. 2016. pNEB193-derived suicide plasmids for gene deletion and protein expression in the methane-producing archaeon, *Methanosarcina acetivorans*. *Plasmid* 84–85:27–35.
